# Hypoxia-driven angiogenesis and metabolic reprogramming in vascular tumors

**DOI:** 10.3389/fcell.2025.1572909

**Published:** 2025-05-15

**Authors:** Lu Liu, Jiayun Yu, Yang Liu, Liang Xie, Fan Hu, Hanmin Liu

**Affiliations:** ^1^ Department of Pediatric Pulmonology and Immunology, West China Second University Hospital, Sichuan University, Chengdu, China; ^2^ Key Laboratory of Birth Defect and Related Diseases of Women and Children (Sichuan University), Ministry of Education, Chengdu, Sichuan, China; ^3^ NHC Key Laboratory of Chronobiology (Sichuan University), Chengdu, China; ^4^ The Joint Laboratory for Lung Development and Related Diseases of West China Second University Hospital, Sichuan University and School of Life Sciences of Fudan University, West China Institute of Women and Children’s Health, West China Second University Hospital, Sichuan University, Chengdu, China; ^5^ Sichuan Birth Defects Clinical Research Center, West China Second University Hospital, Sichuan University, Chengdu, China; ^6^ Department of Radiotherapy, Cancer Center, State Key Laboratory of Biotherapy, West China Hospital, National Clinical Research Center for Geriatrics, Sichuan University, Chengdu, China; ^7^ Department of Pediatric Cardiology, West China Second University Hospital, Sichuan University, Chengdu, Sichuan, China; ^8^ Department of Pediatric Pulmonology and Immunology, WCSUH -Tianfu·Sichuan Provincial Children’s Hospital, Sichuan University, Meishan, China

**Keywords:** vascular tumors, hypoxia, HIF-1α, angiogenesis, metabolic reprogramming, tumor microenvironment

## Abstract

Hypoxia is a hallmark of the tumor microenvironment (TME), and it plays a crucial role in the occurrence and progression in vascular tumors. Under hypoxic conditions, hypoxia-inducible factor 1-alpha (HIF-1α) is stabilized, inducing changes in the expression of various target genes involved in angiogenesis, metabolism, and cell survival. This includes the upregulation of pro-angiogenic factors like VEGF, which promotes the formation of dysfunctional blood vessels, contributing to the worsening of the hypoxic microenvironment. At the same time, hypoxia induces a metabolic shift toward glycolysis, even in the presence of oxygen, supporting tumor cell survival and proliferation by providing necessary energy and biosynthetic precursors. This review discusses the molecular mechanisms by which hypoxia regulates angiogenesis and metabolic reprogramming in vascular tumors, highlighting the intricate link between these processes, and explores potential therapeutic strategies to target these pathways in order to develop effective treatment strategies for patients.

## 1 Introduction

Vascular tumors are neoplasms composed of endothelial cells (ECs), characterized by abnormal proliferation and abnormal vessel formation (Folpe, 2024; Tortorelli et al., 2024). These tumors are typically associated with excessive angiogenesis or abnormal vessel dilation, and they can affect various tissues, including the skin, soft tissues, and internal organs (Mansfield et al., 2020; Urban and Williams, 2024). Based on histopathological features and clinical presentation, vascular tumors are classified into benign, locally aggressive or borderline, and malignant categories. The International Society for the Study of Vascular Anomalies (ISSVA) classification provides a comprehensive stratification of these tumors, as summarized in Table 1 (Kunimoto et al., 2022). Vascular tumors exhibit significant heterogeneity, with the most common type being infantile hemangiomas (IH), which have an incidence rate of approximately 5%–10% (Chen et al., 2019; Nguyen et al., 2020; Eisenstein, 2023), whereas malignant forms such as angiosarcoma (AS) are relatively rare (Dufresne et al., 2023; An et al., 2024). Regardless of whether they are benign or malignant, vascular tumors can have severe impacts on the health of the host, leading to disfigurement, deformities, functional impairments, and even life-threatening conditions. Although existing therapeutic approaches can alleviate some symptoms, the treatment outcomes for most types remain limited, and complete cure is often difficult to achieve, for example, in kaposiform hemangioendothelioma (Ji et al., 2020; Qiu et al., 2025).

**TABLE 1:** ISSVA classification of vascular tumors.

Classification of vascular tumors		
Benign	Locally aggressive or borderline	Malignant
Infantile hemangioma Congenital hemangioma Tufted angioma Spindle-cell hemangioma Epithelioid hemangioma Pyogenic granuloma Others	Kaposiform hemangioendothelioma Retiform hemangioendothelioma Papillary intralymphatic angioendothelioma (PILA), Dabska tumor Composite hemangioendothelioma Pseudomyogenic hemangioendothelioma Polymorphous hemangioendothelioma Hemangioendothelioma not otherwise specified Kaposi sarcoma Others	Angiosarcoma Epithelioid hemangioendothelioma Others

In recent years, the role of hypoxia in tumorigenesis has gained increasing attention. Hypoxia, a prevalent characteristic of tumors, exerts a pivotal influence on tumor progression by modulating a variety of cellular processes, including angiogenesis, metabolic reprogramming, and immune evasion (Wicks and Semenza, 2022; Chen et al., 2023). The hypoxic tumor microenvironment triggers the activation of critical transcriptional regulators, particularly hypoxia-inducible factors (HIFs), which orchestrate the expression of genes involved in cellular adaptation to oxygen deprivation (Luo et al., 2022). In the context of vascular tumors, numerous studies have highlighted the crucial role of hypoxia in driving angiogenesis and metabolic reprogramming, a process essential for tumor growth and progression. This review will focus on the pathogenic mechanisms of hypoxia in vascular tumors, with particular emphasis on hypoxia-driven angiogenesis and metabolic reprogramming. It aims to provide valuable insights and references for researchers and clinicians in the field, with the goal of advancing the development of targeted therapies for vascular tumors.

## 2 Vascular tumors

### 2.1 Infantile hemangioma

IH, the most common benign vascular tumor in infants, is more frequently observed in Caucasians, females, and preterm infants (Ding et al., 2020; Rodríguez Bandera et al., 2021). It typically presents as red or purple masses on the skin or subcutaneous tissue, varying in size, shape, and number, with some cases showing raised vascular lesions. Based on morphological characteristics, IH can be classified into superficial, deep, or mixed types (Forbess Smith et al., 2017). IH generally begins to grow rapidly during infancy, exhibiting a growth pattern characterized by alternating phases of proliferation and regression (Sebaratnam et al., 2021). During the proliferative phase, ECs proliferate significantly within a short period, followed by the regression phase, during which proliferating ECs are gradually replaced by fibrofatty tissue (Greenberger and Bischoff, 2011). Most IH enters a natural regression phase around 1 year of age, with complete regression typically occurring by 3.5 years (Bauland et al., 2011; Couto et al., 2012). In contrast to superficial lesions, deeper IH is often diagnosed later and has a longer growth cycle (Krowchuk et al., 2019). While most IH does not require aggressive treatment, a small proportion may lead to complications during the proliferative phase, such as ulceration, bleeding, and pain (Mitra et al., 2024). In some cases, disfigurement, functional impairment, organ involvement, and even life-threatening complications may arise (Baselga et al., 2016; Krowchuk et al., 2019). IH has been associated with PHACE syndrome, which encompasses a range of clinical manifestations, including posterior fossa brain malformations, arterial anomalies, cardiovascular defects, ocular abnormalities, and cutaneous hemangiomas (Forde et al., 2017). Additionally, IH is linked to LUMBAR syndrome, characterized by lower body IH, cutaneous defects, urogenital malformations, ulceration, myelopathy, bony deformities, anorectal anomalies, arterial anomalies, and renal abnormalities (Yu et al., 2017b). These syndromic associations can significantly impact the quality of life in affected children. Treatment methods for IH include pharmacological therapy (such as propranolol treatment), laser therapy, and surgical excision, aimed at preventing permanent disfigurement due to incomplete regression and associated complications (Krowchuk et al., 2019; Sebaratnam et al., 2021).

### 2.2 Kaposiform hemangioendothelioma

KHE is a rare, locally invasive vascular tumor primarily seen in infants and children, with an incidence of approximately 0.7 per 1,000,000 newborns (Fernández et al., 2009; Croteau et al., 2013). Its name is derived from the “pseudopalisade” structure observed in ECs under the microscope, resembling Kaposi Sarcoma (KS) (Lyons et al., 2004). In 1993, Zukerberg et al. first classified KHE as a distinct entity from IH(Zukerberg et al., 1993). KHE presents with a variety of clinical manifestations. Cutaneous lesions often appear purple and typically grow through invasive infiltration, commonly affecting superficial or deep soft tissues of the limbs and torso (Ji et al., 2020). Another hallmark feature is the Kasabach-Merritt phenomenon (KMP), which is characterized by thrombocytopenia and coagulation abnormalities, often leading to bleeding tendencies such as skin purpura and internal hemorrhage (Kasabach and Merritt, 1940; Croteau et al., 2013; Ji et al., 2018). Unlike KS, KHE is not associated with viral infections. Current studies indicate that KHE is closely related to somatic mutations in the *GNA14* gene (c.614A > T, p.Gln205Leu) (Lim et al., 2016). Treatment options include surgical excision, interventional embolization, and systemic therapies such as rapamycin and vincristine. For cases complicated by KMP, anticoagulant therapy and blood product transfusions are typically used (Drolet et al., 2013; Liu et al., 2016). However, the rarity of KHE and the heterogeneity of its treatment regimens present significant challenges in management.

### 2.3 Kaposi sarcoma

Kaposi sarcoma (KS) is a vascular-originating, locally invasive or borderline tumor, first described by the Italian physician Moritz Kaposi in 1872 (Kaposi, 1872). It arises from the abnormal proliferation of ECs and presents as purple plaques, nodules, or masses, typically involving the skin, lymph nodes, and internal organs, such as the gastrointestinal tract and lungs (Cesarman et al., 2019). KS is classified into four main types: 1) Classic; 2) Endemic; 3) HIV/AIDS-associated; and 4) Iatrogenic (Curtiss et al., 2016). The classic type is commonly seen in elderly men of Mediterranean or Jewish descent, progresses slowly, and is predominantly located on the skin of the lower limbs and face (Curtiss et al., 2016). The endemic type is more common among young men and children in sub-Saharan Africa, progresses more rapidly, and is often associated with lymphadenopathy (Stefan, 2015; El-Mallawany et al., 2016). HIV/AIDS-associated KS is closely related to HIV infection and is commonly observed in men who have sex with men (Gottlieb et al., 1981). It typically presents with skin, lymph node, and visceral involvement, may progress rapidly, and is associated with immune suppression. Iatrogenic KS primarily occurs in organ transplant recipients but can also result from chemotherapy or other immunosuppressive treatments, typically affecting the skin with some visceral involvement (Siegel et al., 1969; Grulich and Vajdic, 2015). In 1994, Kaposi’s sarcoma-associated herpesvirus (KSHV), also known as human herpesvirus 8 (HHV-8), was identified as the causative agent of KS(Chang et al., 1994). The interplay between KSHV infection and host immune impairment contributes to the pathogenesis of KS. The treatment for KS depends on the type, rate of progression, and the immune status of the patient (Cesarman et al., 2019). Therapeutic approaches include antiviral therapy, local treatments (such as laser therapy or cryotherapy), chemotherapy, and immunotherapy (Schneider and Dittmer, 2017).

### 2.4 Angiosarcoma

AS is a rare and highly aggressive malignant tumor originating from the ECs of blood vessels or lymphatic vessels, accounting for 1%–2% of all soft tissue sarcomas (Young et al., 2010; Cao et al., 2019). It commonly occurs in elderly individuals, with an average age of onset at 73 years, and there is no significant gender bias (Albores-Saavedra et al., 2011; An et al., 2024). Although the exact mechanisms underlying AS are not fully understood, several well-established risk factors include chronic lymphedema (Stewart-Treves syndrome), a history of radiation therapy, and exposure to environmental chemicals such as vinyl chloride, thorium dioxide, and arsenic (Virtanen et al., 2007; Young et al., 2010; Chaudhary et al., 2015; Pereira et al., 2015; Disharoon et al., 2017). AS can develop in almost any soft tissue or visceral organ, with clinical presentations varying depending on the tumor location. The tumor can be classified into types such as cutaneous AS, lymphedema-associated AS, radiation-induced AS, primary breast AS, and soft tissue AS, with cutaneous AS being the most common type, typically affecting the head, neck, and particularly the scalp (Young et al., 2010; Bi et al., 2022). The diagnosis of AS primarily relies on pathological examination, which reveals abnormal, pleomorphic ECs proliferation (Young et al., 2010). In well-differentiated regions, atypical ECs form anastomosing vascular channels resembling normal blood vessels. With tumor progression, the architecture becomes increasingly disorganized, characterized by poorly defined vascular spaces and frequent intraluminal red blood cell accumulation (Ronchi et al., 2020). In poorly differentiated areas, the ECs form sheet-like arrangements, usually exhibiting an epithelial-like morphology, and are often associated with bleeding and necrosis (Marušić and Billings, 2017). AS typically expresses endothelial cell-specific markers, including factor VIII-related antigen (Factor-VIIIRA), CD31, CD34, and vascular endothelial growth factor (VEGF) (Ohsawa et al., 1995). AS is highly malignant, with an overall 5-year survival rate of approximately 35% (Mark et al., 1996; Fury et al., 2005; Fayette et al., 2007). Current treatment options include surgical resection, radiation therapy, chemotherapy, immunotherapy, and targeted therapy (Young et al., 2010; Cao et al., 2019). However, due to the high recurrence and metastatic potential of AS, preventing recurrence and metastasis after treatment remains a significant clinical challenge.

## 3 HIFs-mediated signal transduction

### 3.1 Overview of HIFs

**FIGURE 1:**
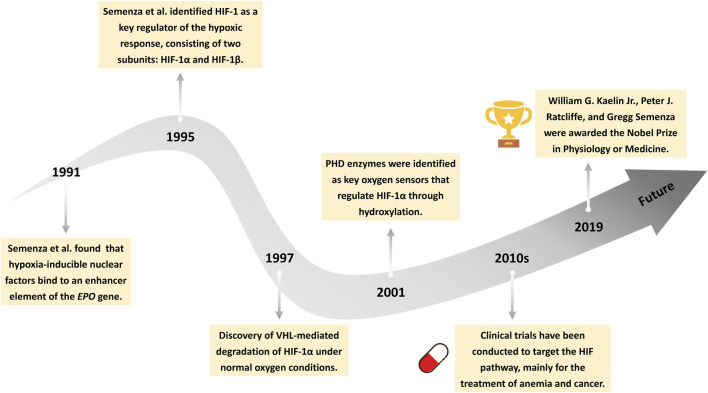
Milestones in the history of HIF signaling. This timeline illustrates key discoveries in the HIFs pathway, from the identification of hypoxia-inducible nuclear factors (1991), to the cloning of HIF-1 (1995), the elucidation of von Hippel-Lindau (VHL)-mediated degradation (1997), the identification of prolyl hydroxylases (PHD) enzymes (2001), the initiation of clinical trials (2010s), and the awarding of the 2019 Nobel Prize in Physiology or Medicine.

Oxygen homeostasis is vital for eukaryotic survival. Sensing and regulating hypoxia is crucial for this process. HIFs are transcription factors that enable cells to adapt to changes in oxygen levels. Significant progress has been made in the study of the HIF pathway over the past three decades (Figure 1). In 1991, Semenza et al. (1991) found that hypoxia-inducible nuclear factors bind to an enhancer element of the erythropoietin *(EPO)* gene, providing the first molecular insight into oxygen-dependent gene regulation. Subsequently, in 1995, the same group successfully purified and characterized HIF-1 as a heterodimeric transcription factor composed of two subunits: HIF-1α and HIF-1β (Wang and Semenza, 1995; Wang et al., 1995). In the following years, the precise regulatory mechanisms of HIF signaling were progressively elucidated. In 1996, HIF-1α was found to undergo oxygen-dependent degradation mediated by the von Hippel–Lindau (VHL) tumor suppressor protein under normoxic conditions (Iliopoulos et al., 1996). Loss of VHL function was shown to stabilize HIF-1α, thereby promoting its accumulation and transcriptional activity (Maxwell et al., 1999). In 2001, prolyl hydroxylase (PHD) enzymes were identified as the key oxygen sensors responsible for hydroxylating specific proline residues on HIF-1α, enabling its recognition and subsequent degradation via the VHL pathway (Jaakkola et al., 2001). More recently, research has shown that the molecular mechanisms of HIFs are closely associated with diseases such as cancer (Semenza, 2003), anemia (Haase, 2013), inflammation (McGettrick and O’Neill, 2020), and cardiovascular diseases (Liu et al., 2020). In the 2010s, clinical trials targeting the HIF signaling pathway were initiated, with a primary focus on therapeutic applications in anemia and cancer (Semenza, 2019; Liu et al., 2024). William Kaelin, Peter Ratcliffe, and Gregg Semenza were awarded the 2019 Nobel Prize in Physiology or Medicine for their groundbreaking contributions to the discovery of how cells sense and adapt to changes in oxygen availability (Ledford and Callaway, 2019).

The HIFs transcription factor family currently includes HIF-1, HIF-2, and HIF-3 (Korbecki et al., 2021). HIF-1 and HIF-2 primarily mediate the transcription of hypoxia-inducible genes, whereas HIF-3 not only activates gene expression but also inhibits the activity of HIF-1 and HIF-2 (Tanaka et al., 2009; Zhang et al., 2014). The structure of HIFs is complex, consisting of two subunits: HIF-α and HIF-β (Wang et al., 1995). HIF-β is a stable subunit, ubiquitously expressed in various cells, and is insensitive to changes in oxygen levels. The HIF-β subunit, also known as the aryl hydrocarbon receptor nuclear translocator (ARNT), is encoded by the *ARNT1*, *ARNT2*, and *ARNT3* genes (Wang et al., 1995; Dengler et al., 2014). In contrast, the HIF-α subunit is primarily regulated by oxygen levels. Three isoforms of the HIF-α subunit have been identified in humans and mammals: HIF-1α, HIF-2α, and HIF-3α (Zhao et al., 2024). Among these, HIF-1α is the most widely studied and is expressed in most human cells, while HIF-2α is expressed in specific tissues and cell types, such as the lungs, kidneys, and liver. HIF-3α is predominantly expressed in cardiac, renal, and pulmonary epithelial cells (Yang et al., 2015).

### 3.2 Regulation of HIFs

**FIGURE 2:**
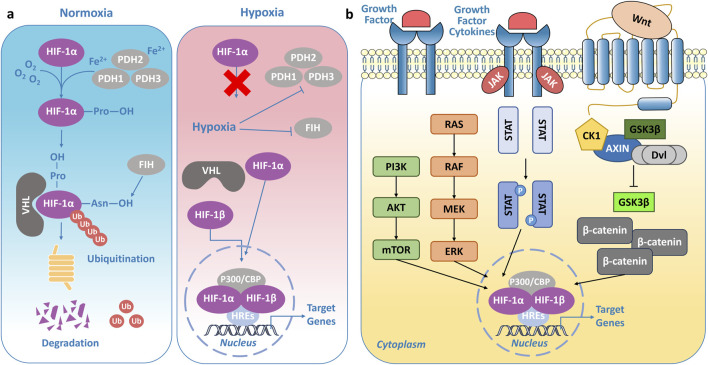
Regulation of HIFs. **(A)** Under normoxic conditions, HIF-1α is hydroxylated by prolyl hydroxylases (PHDs), promoting its recognition by the von Hippel-Lindau (VHL) E3 ubiquitin ligase complex and subsequent degradation via the proteasome. In contrast, under hypoxic conditions, limited oxygen availability inhibits PHD and FIH activity, leading to HIF-1α stabilization and activation of hypoxia-related transcriptional programs. **(B)** Regulatory network of signaling pathways controlling HIF activity.

HIFs are critical transcription factors that govern cellular responses to oxygen levels. The activity of HIFs, particularly HIF-1α, is tightly regulated by oxygen availability to ensure that the hypoxic response is activated only when required. Under normoxic conditions, HIF-1α is rapidly degraded to prevent the activation of hypoxia-responsive genes. In normal cells, HIF-1α is predominantly degraded through the ubiquitin-proteasome pathway (Figure 2A) (Lee et al., 2004). The oxygen-dependent degradation domain (ODDD) of HIF-1α undergoes hydroxylation at proline residues (such as Pro-402 and Pro-564) by PHDs, including PHD1, PHD2, and PHD3, which are iron- and α-ketoglutarate-dependent enzymes (Salceda and Caro, 1997; Ivan et al., 2001; Jaakkola et al., 2001). Subsequently, Factor Inhibiting HIF (FIH) hydroxylates Asn803 of HIF-1α, preventing its interaction with coactivators like p300/CBP (Mahon et al., 2001; Lando et al., 2002). This modification promotes recognition by the VHL E3 ubiquitin ligase complex, leading to the ubiquitination of the HIF-1α subunit, which is then degraded by the 26S proteasome (Salceda and Caro, 1997; Hon et al., 2002). Under hypoxic conditions, due to limited oxygen supply, the activity of PHDs and FIH is significantly reduced (Freedman et al., 2002; Majmundar et al., 2010). As a result, proline and asparagine residues in HIF-1α cannot undergo hydroxylation, leading to the accumulation of HIF-1α in the cytoplasm. It then translocates to the nucleus. In the nucleus, stable HIF-1α dimerizes with HIF-1β (ARNT) and, through interaction with coactivators like p300/CREB-binding protein (CBP), regulates gene expression by binding to hypoxia-responsive elements (HREs) (Wu et al., 2015; Bao et al., 2021). This forms a transcriptionally active complex that induces the robust expression of downstream target genes (Figure 2A).

What’s more, HIFs are regulated by multiple signaling pathways (Figure 2B). HIF-1α is a downstream target of the mechanistic target of rapamycin (mTOR) signaling pathway and is tightly regulated by the phosphoinositide 3-kinase (PI3K)/protein kinase B (Akt)/mTOR axis (Shimobayashi and Hall, 2014). In tumors, loss-of-function mutations in the tumor suppressor gene *PTEN* impair its negative regulatory effects on PI3K signaling, leading to decreased PI3K degradation and sustained phosphorylation-driven activation of Akt and mTOR (Steelman et al., 2004; Chen et al., 2018). This dysregulated signaling cascade ultimately facilitates the transcriptional upregulation of *HIF-1α* mRNA. The mitogen-activated protein kinase/extracellular signal-regulated kinase (MAPK/ERK) pathway represents another critical signaling cascade regulating HIF-1α (Wan and Wu, 2016). Studies have demonstrated that MAPK signaling can activate the HIF-1α pathway by modulating the p300/CBP coactivator complex, thereby enhancing its transcriptional activity (Sang et al., 2003). Additionally, ERK directly regulates HIF-1α transcriptional activity, further amplifying its function (Sang et al., 2003). The Janus kinase/signal transducer and activator of transcription (JAK/STAT) signaling pathway plays a pivotal role in regulating HIF-1α expression and stability through multiple mechanisms. Specifically, the JAK/STAT3 axis directly promotes HIF-1α gene transcription, increasing its mRNA levels (Xu et al., 2005; Zhao and Qin, 2019; Zhao et al., 2019). Moreover, STAT3 directly interacts with HIF-1α, competitively interfering with pVHL-mediated ubiquitination, thereby attenuating its proteasomal degradation and enhancing its stability (Jung et al., 2008). Interestingly, a bidirectional regulatory mechanism exists between the Wnt (Wingless/Integrated)/β-catenin signaling pathway and HIF-1α (Liu et al., 2015; 2021). Wnt/β-catenin can indirectly modulate HIF-1α expression through the PI3K/Akt and ERK/MAPK pathways (Lau et al., 2011). In addition to the aforementioned signaling pathways, the Notch and nuclear factor-kappa B (NF-κB) pathways also contribute to the regulation of HIFs, integrating hypoxia signaling with essential cellular processes such as differentiation, proliferation, and metabolism (Malekan et al., 2021).

### 3.3 Physiological functions of HIFs

HIFs regulate a variety of biological processes that help cells and tissues adapt to hypoxic environments. These processes include angiogenesis, metabolic reprogramming, cell survival and apoptosis, erythropoiesis, immune response regulation, and tissue repair and regeneration (Luo et al., 2022). Both HIF-1α and HIF-2α play critical roles in oxygen homeostasis but differ in their tissue-specific expression and target genes. HIF-1α is widely expressed across most cell types and primarily regulates immediate hypoxic responses, controlling the expression of over 100 downstream genes (Yang et al., 2015; Choi, 2017). Key target genes regulated by HIF-1α include *VEGF* for angiogenesis, *GLUT-1* (glucose transporter 1) for enhanced glucose uptake, and *LDHA* (lactate dehydrogenase A) for promoting anaerobic glycolysis (Firth et al., 1994). Additionally, HIF-1α regulates genes involved in cell survival, such as *BCL-2*, and apoptosis, such as BCL2 Interacting Protein 3 (*BNIP3*) (Manalo et al., 2005). In the immune response, HIF-1α modulates the expression of various cytokines and chemokines, which recruit immune cells to sites of infection or injury (Eltzschig and Carmeliet, 2011; Palsson-McDermott et al., 2015; Codo et al., 2020). Moreover, HIF-1α plays a critical role in tissue repair and regeneration by promoting angiogenesis, metabolic adaptation, and the repair of damaged tissues (Konieczny et al., 2022; Wang et al., 2023). In contrast, HIF-2α is expressed in specific tissues, such as the lungs, kidneys, and liver, and plays a key role in regulating erythropoiesis and cell differentiation (Wiesener et al., 2003).

## 4 Hypoxic signaling in vascular tumors

### 4.1 Hypoxia as a common feature of tumors

Hypoxic regions are commonly observed in a wide range of human tumors. As early as the 1990s, Höckel et al. employed a computerized pO_2_ histography system to assess oxygen levels in cervical and breast cancers, identifying marked reductions in pO_2_ (Höckel et al., 1991; Vaupel et al., 1991). Vaupel et al. also assessed the partial oxygen pressure (pO_2_) in several cancer types and reported that the median pO_2_ in breast, cervical, and head/neck cancers was 10 mmHg (approximately 1.4% O_2_) (Vaupel et al., 2007). Notably, approximately 25% of the measurements were below 2.5 mmHg, with some approaching zero, indicating the presence of a severely hypoxic microenvironment within these tumors. Furthermore, in experimental animal models, whether syngeneic or xenograft tumor transplants are used, a consistent reduction in tumor tissue pO_2_ has been observed (Günther et al., 1972; Vaupel et al., 1987). Höckel et al. further discovered that tumor hypoxia (reduced pO_2_) is associated with poor prognosis in primary cervical cancer (Höckel et al., 1993; Höckel et al., 1996).

Tumor hypoxia is a key regulator of HIF-1α and HIF-2α expression, driving their upregulation in response to oxygen deprivation. Elevated levels of one or both proteins have been widely documented in various human tumors. Notably, in clear cell renal cell carcinoma (ccRCC), deletions or mutations of the *VHL* gene impair the proteasomal degradation of HIF-1α and HIF-2α, leading to their aberrant accumulation even under normoxic conditions (Schödel et al., 2016; Hsieh et al., 2017). Similarly, in breast cancer, HIF-1α expression is significantly upregulated and exhibits a heterogeneous distribution, primarily localized to viable cancer cells surrounding necrotic regions (Gruber et al., 2004; Jin et al., 2016). Additionally, HIF-1α expression has been detected in certain stromal cells, ECs, and tumor-associated macrophages (TAMs) (Bos et al., 2003; Dales et al., 2005). Furthermore, some studies have also reported increased expression of HIF-2α, suggesting a potential role in breast cancer progression (Leek et al., 2002; Helczynska et al., 2008). Pseudopalisades, a characteristic pathological feature of glioblastoma (GBM), are driven by HIF-1α upregulation in hypoxic tumor cells, which promotes their migration away from regions of vascular occlusion and necrosis (Rong et al., 2006; Ji et al., 2013). Beyond these, HIF-1α and HIF-2α upregulation has also been reported in multiple other solid tumors, including bladder cancer, hepatocellular carcinoma, colorectal cancer, and sarcoma (Nordsmark et al., 2001; Theodoropoulos et al., 2004; 2005; Morine et al., 2011; Tang et al., 2018). Interestingly, increased expression of HIFs has been observed in hematologic malignancies, such as leukemia, lymphoma, and multiple myeloma (Evens et al., 2010; Deeb et al., 2011; Frolova et al., 2012; Borsi et al., 2015). Taken together, Hypoxia-induced HIFs upregulation represents a fundamental mechanism in tumor pathophysiology, broadly contributing to the adaptive and pathological processes.

### 4.2 Causes of hypoxia in tumors

Hypoxia is a hallmark of the tumors, resulting from a combination of structural and metabolic factors that limit oxygen availability. Firstly, the uncontrolled proliferation and rapid growth of tumor cells require large amounts of oxygen and nutrients. When oxygen demand exceeds supply, hypoxic regions develop within the tumor (Vander Heiden et al., 2009). Although the expression of erythropoietin (EPO) and angiogenic factors is induced under hypoxic conditions, the newly formed blood vessels are often disorganized and irregular, leading to dysfunctional vessels that fail to provide adequate perfusion, which in turn exacerbates blood flow stasis and worsens oxygen deficiency (Baluk et al., 2005; Nagy et al., 2010). Secondly, the diffusion distance of oxygen is limited, and hypoxia is commonly observed in tissue areas more than 100–200 µm from functional blood vessels (Dachs et al., 1997). What’s more, the elevated interstitial pressure within the tumor, caused by tumor cell proliferation and extracellular matrix alterations, compresses blood vessels, further restricting blood flow. This mechanical obstruction makes it even more difficult for blood to reach the core regions of the tumor, leading to widespread hypoxia (Padera et al., 2004). Aside from the factors mentioned above, several additional mechanisms also contribute to tumor hypoxia. Tumor cells typically exhibit metabolic reprogramming (the Warburg effect), wherein they preferentially rely on anaerobic glycolysis for energy production, even under normoxic conditions (Warburg, 1925). This metabolic pathway not only leads to the accumulation of large amounts of lactate but also causes a local decrease in pH within the tumor region, further exacerbating the hypoxic condition (Jaworska et al., 2023). Additionally, a reduction in the number of red blood cells or a decrease in the oxygen-carrying capacity of hemoglobin may also result in a reduced supply of oxygen to the tumor tissue, thus intensifying the hypoxia (Vaupel et al., 2003). In conclusion, tumor hypoxia results from a combination of structural and metabolic factors, including rapid tumor growth, dysfunctional blood vessels, limited oxygen diffusion, and metabolic reprogramming, all of which contribute to the establishment of hypoxic regions within tumors.

### 4.3 Hypoxic signaling in infantile hemangioma

IH is closely associated with hypoxia. Clinical studies have shown that IH is related to several known risk factors, including prematurity, low birth weight, and placental insufficiency (such as preeclampsia and placental hypoxia) (de Jong et al., 2016). Studies indicate that approximately 30% of extremely low birth weight infants (weighing <1000 g) develop IH (Amir et al., 1986). Research by Colonna et al. (2010) and López Gutiérrez et al. (2007) has demonstrated that infants with IH have a significantly higher incidence of placental hypoxia compared to controls. Furthermore, infants born after pregnancies complicated by preeclampsia or placental abnormalities also show a higher incidence of IH (Haggstrom et al., 2007). Prior to the development of IH lesions, infants often present with early skin changes, such as pallor, blanching, ecchymosis, or capillary dilation (Drolet and Frieden, 2010). These early skin manifestations are likely caused by vascular constriction leading to tissue ischemia, resulting in “anemic spots” or “low blood flow areas” (Herbert et al., 2011). In summary, the clinical evidence suggests that the development of IH is closely linked to hypoxia.

Both Xia et al. (2017) and Kleinman et al. (2007) have found that HIF-1α is significantly upregulated in proliferating infantile hemangioma (IH) tissue sections. In these proliferative tumor sections, HIF-1α is predominantly expressed in the nuclei of endothelial and stromal cells. In contrast, HIF-1α expression is not observed in tissues during the involution phase. The upregulation of HIF-1α plays a key role in the progression of IH, promoting the proliferation and migration of hemangioma endothelial cells (HemECs) (Xiang et al., 2024). Additionally, HIF-1α induces the expression of target genes, such as *VEGF*, *GLUT-1*, Matrix Metalloproteinase 9 (*MMP-9*), Stromal Cell-Derived Factor 1 alpha (*SDF-1α*), and *BNIP3*, further driving tumor angiogenesis, metabolic reprogramming, and anti-apoptotic processes (North et al., 2000; Kleinman et al., 2007; Janmohamed et al., 2015; van Vugt et al., 2017; Wu et al., 2021). However, an integrated microarray analysis revealed that the hypoxic environment in IH is primarily regulated by HIF-2α, rather than HIF-1 (Gomez-Acevedo et al., 2020). Drolet and Frieden (2010) suggest that the formation of IH is a response to a hypoxic environment, representing a homeostatic attempt to normalize hypoxic tissue, a process induced by HIFs. Overall, hypoxia and HIFs play a crucial role in the progression of IH.

### 4.4 Hypoxic signaling in kaposiform hemangioendothelioma

KHE is a rare orphan disease, and consequently, research on its pathophysiology, molecular mechanisms, and therapeutic strategies remains limited, with much of the existing literature based on case reports. However, evidence suggests the presence of hypoxic signaling in KHE. Elevated expression of HIF-1α has been observed in the lesions of KHE patients (Ji et al., 2020; Li et al., 2024). In a study by Li et al., a 3D spheroid model of KHE was established using the EOMA, utilizing a rotary cell culture system (RCCS) to replicate the tumor microenvironment. The results showed that HIF-1α expression was significantly upregulated both in the EOMA spheroids and in the EOMA xenograft mouse model (Li et al., 2024). This upregulation is likely due to the activation of the PI3K/Akt/mTOR signaling pathway in KHE ECs, leading to the phosphorylation and overactivation of Akt1, mTORC1 and mTORC2, which in turn enhances the translation of downstream HIF-1α (Ji et al., 2020; Wang et al., 2020; Qiu et al., 2025). Furthermore, in KHE, HIF-1α induces the expression of pro-angiogenic genes, including Vascular Endothelial Growth Factor C (*VEGFC*) and Vascular Endothelial Growth Factor Receptor 3 (*VEGFR3*) (Saito et al., 2009; Cohen et al., 2022). In contrast to IH, *GLUT-1* immunostaining was negative in all KHE cases (van Vugt et al., 2017; Johnson et al., 2018).

### 4.5 Hypoxic signaling in kaposi sarcoma

There is substantial evidence indicating that hypoxia and HIFs play a central role in the pathogenesis of KS and KSHV infection. Interestingly, Kaposi himself noted in his initial description that KS predominantly affects the feet and lower extremities, areas that are typically under low oxygen conditions (Kaposi, 1872). A study by Long et al. on tissue samples from 245 HIV-positive patients revealed that HIF-1α expression was present in KS biopsies at various stages, with its levels continuously increasing throughout tumor progression (Long et al., 2009). Research by Catrina et al. further demonstrated that both HIF-1α and HIF-2α were expressed in KS biopsies, with expression detected across all stages of the tumor, peaking in the late-stage nodular phase (Catrina et al., 2006). The activation of HIFs also leads to the upregulation of downstream target genes, such as VEGF, Bcl-2, and Mcl-1, further contributing to the KS progression (Catrina et al., 2006; Long et al., 2009).

In the context of KSHV infection, hypoxia also plays a crucial role in the virus’s lytic replication, further facilitating viral spread and disease progression. As a γ-herpesvirus, KSHV’s genome contains several HREs, which, through various mechanisms, promote the upregulation of HIFs (Aneja and Yuan, 2017; Davis et al., 2023). Known HREs include those in ORF34-ORF37 and RTA, with the RTA promoter primarily responding to HIF-2α, while the ORF34 promoter responds to both HIF-1α and HIF-2α, leading to the activation of KSHV lytic replication (Cai Q. et al., 2006; Haque et al., 2006; Aneja and Yuan, 2017). Additionally, the ORF37 gene of KSHV encodes a shutoff ribonuclease (SOX) that degrades mRNA and suppresses the expression of most host genes, though it does not affect the mRNA levels of HIF-1α (Glaunsinger and Ganem, 2004). The latent nuclear antigen (LANA) encoded by ORF73, which is expressed during the latent phase of infection, forms a complex with HIF-1α and recruits chromatin remodeling enzyme KAP1 to the RTA promoter region, further regulating the initiation of lytic replication (Cai Q. et al., 2006; Cai et al., 2013). These mechanisms collectively highlight the critical role of hypoxia in KSHV infection and its potential involvement in the regulation of viral latency and reactivation.

### 4.6 Hypoxic signaling in angiosarcoma

In AS, the presence of hypoxic signaling has been confirmed by several studies. Due to the rarity of AS, most relevant research is concentrated in case reports. Research by Maeda-Otsuka et al. (2019) found that HIF-1α expression in AS tissues was significantly upregulated and exhibited heterogeneity, with stronger expression of HIF-1α in tumor cells distant from blood vessels and weaker expression in cells closer to the vasculature. Additionally, Al-Salam et al. (2012) observed high expression of HIF-1α in tissue sections of breast AS. Studies have also indicated upregulation of HIF-1α and HIF-2α expression in retroperitoneal AS, accompanied by the induction of hypoxia-responsive genes (Rathmell et al., 2004). In sporadic cutaneous AS, 3 out of 18 cases demonstrated high HIF-1α expression (Abedalthagafi et al., 2010). Further investigations revealed that downstream genes of HIF-1α, including *VEGF*, Vascular Endothelial Growth Factor Receptor 2 (*VEGFR2*), and *GLUT-1*, were highly expressed in both AS tissue sections and cell lines (Al-Salam et al., 2012; Hoshina et al., 2013; van Vugt et al., 2017). Notably, elevated GLUT-1 expression was significantly associated with high histological grading and was considered an independent prognostic factor (Smeland et al., 2012). Furthermore, AS patients with strong HIF-1α positivity were typically younger and had a higher incidence of lymph node and organ metastasis, emphasizing the clinical significance of hypoxic signaling in AS (Maeda-Otsuka et al., 2019).

## 5 Hypoxia-driven angiogenesis in vascular tumors

**FIGURE 3:**
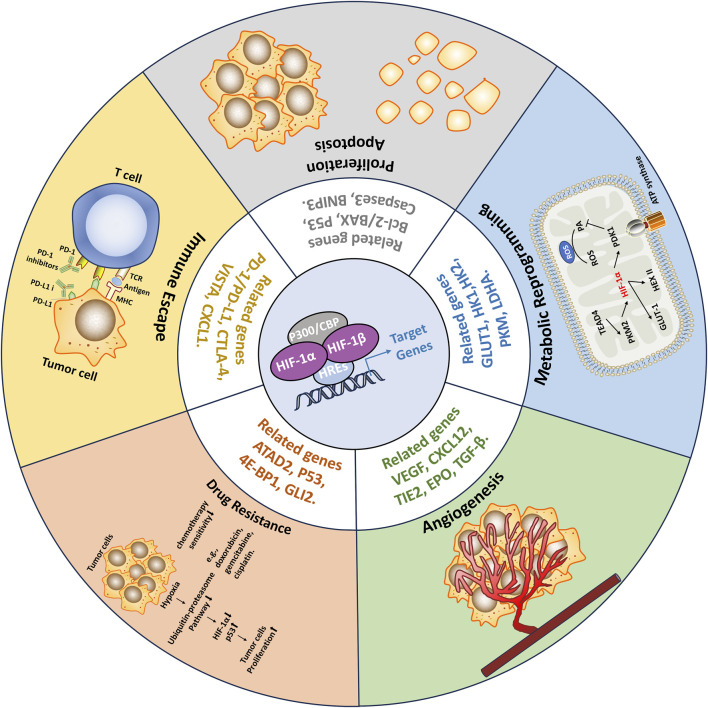
Consequences of HIFs activation in Tumors. Within tumors, due to rapid tumor cell proliferation and incomplete vascular formation, a hypoxic microenvironment (HME) often arises, thereby triggering the activation of HIFs. The activated HIFs regulate the transcription of downstream RNA, which participate in key aspects of cancer progression, including tumor angiogenesis, metabolic reprogramming, tumor cell proliferation, immune evasion, and resistance to therapies. As a result, the tumor is able to survive and continue to progress in hypoxic and other unfavorable conditions.

The tumor microenvironment (TME) refers to the complex milieu surrounding the tumor, comprising various cell types, blood vessels, immune cells, extracellular matrix components, and other molecular factors (Elhanani et al., 2023; Yang et al., 2023). The TME is composed of a diverse range of cellular components, including tumor cells, fibroblasts, immune cells, ECs, and extracellular matrix constituents (Xiao and Yu, 2021). However, within tumors, due to rapid tumor cell proliferation and incomplete vascular formation, a hypoxic microenvironment (HME) often arises, thereby triggering the activation of HIFs. The activated HIFs regulate the transcription of downstream RNA, which participate in key aspects of cancer progression, including tumor angiogenesis, metabolic reprogramming, tumor cell proliferation, immune evasion, and resistance to therapies (Wicks and Semenza, 2022; Zhao et al., 2024; Zhou et al., 2024) (Figure 3). As a result, the tumor is able to survive and continue to progress in hypoxic and other unfavorable conditions.

### 5.1 Hypoxia-driven angiogenesis in tumors

**FIGURE 4:**
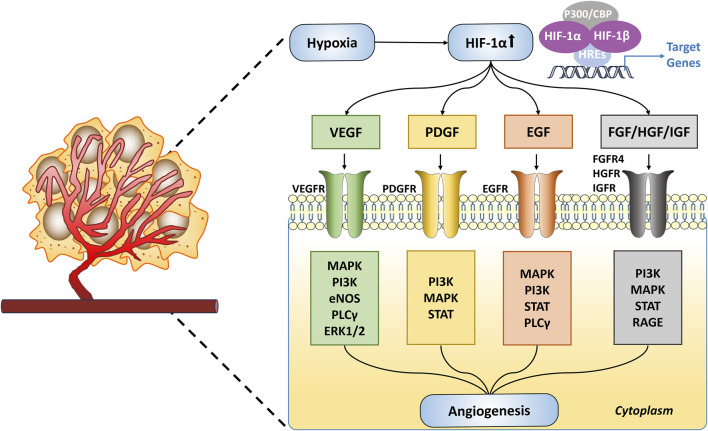
Hypoxia-Driven Angiogenesis in Tumors. Under hypoxic conditions, HIF-1α is upregulated and activates target genes such as VEGF, PDGF, EGF, and FGF/HGF/IGF. These target genes initiate angiogenesis through various signaling pathways.

In tumors, the activation of HIF-1α plays a critical role in angiogenesis. It achieves this by upregulating the expression of various pro-angiogenic growth factors, including VEGF, platelet-derived growth factor (PDGF), and epidermal growth factor (EGF) (Jiang et al., 2020). These growth factors bind to their respective receptors on ECs, activating several signaling pathways that promote ECs proliferation, migration, and the formation of new blood vessels (Figure 4). Specifically, VEGF-A binds to VEGFR2, activating tyrosine kinase, which further stimulates ECs proliferation via the PI3K, MAPK, and ERK1/2 pathways, and increases vascular permeability through the endothelial nitric oxide synthase (eNOS) pathway (Xin et al., 2016; Watari et al., 2020; Wu et al., 2022). Elevated expression of HIF-1α and VEGF is associated with poor prognosis and reduced therapeutic responsiveness (Vageli et al., 2024). Studies have shown that inhibiting VEGF expression can effectively prevent tumor angiogenesis, promote vascular normalization, and suppress tumor growth (Hosaka et al., 2020). The platelet-derived growth factor (PDGF)/platelet-derived growth factor receptor (PDGFR) signaling axis also plays a crucial role in angiogenesis (Li et al., 2022). Phosphorylated PDGFR promotes ECs proliferation primarily via the PI3K-Akt signaling pathway. In animal models, blocking PDGFR-β has been shown to reduce cancer cell growth and migration (Crawford et al., 2009; Wang J.-C. et al., 2019). Additionally, EGF and its receptor epidermal growth factor receptor (EGFR) are pivotal in ECs proliferation, survival, differentiation, and migration (Rajakumar and Pugalendhi, 2023). EGFR and VEGFR often share downstream signaling pathways, and EGFR activation leads to increased VEGFR levels, thus enhancing angiogenesis (Yu et al., 2017a). Human epidermal growth factor receptor 2 (HER2), a member of the EGFR family, is frequently observed in breast cancer and is a well-established therapeutic target (Swain et al., 2023). Besides these factors, other growth factors such as fibroblast growth factor (FGF), hepatocyte growth factor (HGF), insulin-like growth factor (IGF), and transforming growth factor-beta (TGF-β) also play key roles in hypoxia-induced tumor angiogenesis, driving tumor growth and metastasis (Jiang et al., 2020; Magar et al., 2024; Mou et al., 2024). Targeting these signaling pathways holds promise for effectively inhibiting tumor angiogenesis and providing new therapeutic strategies for cancer treatment.

### 5.2 Angiogenesis in vascular tumors

In IH, hypoxia promotes angiogenesis through multiple mechanisms. Zhang et al. demonstrated that under hypoxic conditions, the activation of HIF-1α activates the downstream VEGF/VEGFR2 signaling pathway, promoting blood vessel formation (Zhang et al., 2023). Overexpression of HIF-1α significantly enhanced the angiogenic capacity of HemECs, whereas silencing HIF-1α inhibited the growth of IH xenografts in nude mice. Wu et al. found that Cyr61/CCN1 (cysteine-rich angiogenic inducer 61) is significantly upregulated in the proliferative phase of IH (Wu et al., 2021). As an important extracellular matrix protein, Cyr61/CCN1 promotes angiogenesis under hypoxic conditions. The study revealed that hypoxia significantly increases the production of Cyr61/CCN1 in HemECs in a time-dependent manner, and this upregulation further enhances angiogenesis by inducing VEGFA in HemSCs (hemangioma-derived stem cells). Additionally, Ritter et al. observed the presence of multiple myeloid cells in proliferative IH, which have the ability to differentiate into macrophages, mast cells, and lymphocytes (Ritter et al., 2006). Under hypoxic conditions, these myeloid cells secrete angiogenic factors such as VEGF and IGF-2, further promoting angiogenesis (Ritter et al., 2006; Tan et al., 2015).

In KS, substantial evidence indicates that the stabilization of HIFs promotes the elevation of paracrine angiogenic factors, thereby driving abnormal angiogenesis (Catrina et al., 2006; Shin et al., 2008; Jham et al., 2011). Following KSHV infection of ECs, the transcriptional activity of HIF-1α and HIF-2α is enhanced, even under normoxic conditions. Additionally, both latent and lytic KSHV proteins—such as Latency-Associated Nuclear Antigen 1 (LANA1), viral Interferon Regulatory Factor 3 (vIRF3), and viral G Protein-Coupled Receptor (vGPCR)—upregulate the expression of HIF within the cells. This, in turn, further increases the levels of HIF-related angiogenic factors and cytokines, including VEGF, PDGF, Transforming Growth Factor α (TGFα), Transforming Growth Factor β (TGFβ), Angiopoietin 2 (ANGPT2), and Angiopoietin-Like Protein 4 (ANGPTL4) (Cai Q.-L. et al., 2006; Sodhi et al., 2006; Shin et al., 2008; Jham et al., 2011). These factors bind to their respective receptors on adjacent endothelial cells, activating the mTOR signaling pathway, thereby promoting the upregulation of HIF expression and enhancing HIF-1α signaling, which in turn facilitates the growth of KS tumors (Ma et al., 2010; Jham et al., 2011). Jham et al. found that inhibiting paracrine activation of mTOR was sufficient to suppress the upregulation of HIF within these cells and eliminate their ability to promote tumor formation *in vivo* (Jham et al., 2011).

Due to the rarity of AS, the availability of tumor samples is extremely limited, and thus, molecular studies on angiosarcoma are relatively scarce. Existing research primarily relies on human tumor cell lines, such as ASM and ISO-HAS, which are derived from samples of advanced scalp cutaneous angiosarcoma patients. A study by Yang et al. found that both of these tumor cell lines exhibited significant increases in molecules such as angiogenin, coagulation factor III, FGF1, FGF2, HGF, urokinase-type plasminogen activator (uPA), and VEGF (Young et al., 2014). Furthermore, Azzariti et al. extracted primary cell lines from radiation-induced breast angiosarcoma and, after 48 h of culture, detected the expression of VEGFR2 and VEGFR1 in AS cells, with a higher expression level of VEGFR2 (Azzariti et al., 2014). After treatment with Bevacizumab (anti-VEGF antibody), VEGF levels remained low for 3 days post-treatment, showing a better response than tyrosine kinase inhibitors (TKI). Therefore, anti-angiogenic therapy shows promising clinical potential in the treatment of AS.

## 6 Metabolic reprogramming in vascular tumors

### 6.1 Metabolic reprogramming in tumors

**FIGURE 5:**
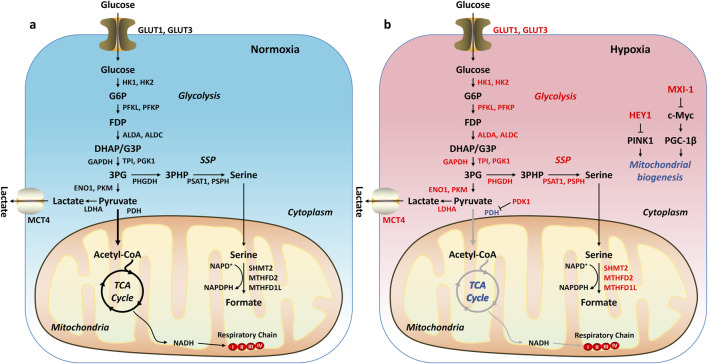
Metabolic Reprogramming in Tumors. **(A)** Under normxia, glucose is broken down into pyruvate through glycolysis. The pyruvate is then converted into acetyl-CoA, which enters the tricarboxylic acid cycle (TCA) to produce the electron donor NADH. These electrons are transferred through the mitochondrial respiratory chain and ultimately passed to oxygen (O_2_), generating adenosine triphosphate (ATP) through oxidative phosphorylation. **(B)** Under hypoxia, HIF-1α is activated, regulating downstream target genes and leading to an increase in glycolysis and/or a decrease in oxidative phosphorylation. Genes or pathways highlighted in red: upregulated by HIF-1α. Genes or pathways highlighted in blue: downregulated by HIF-1α.

Under normal physiological conditions, the body’s energy supply primarily relies on oxidative phosphorylation (OXPHOS). Glucose is broken down into pyruvate through glycolysis, and then pyruvate is converted into acetyl-CoA, which enters the tricarboxylic acid cycle (TCA) to produce the electron donor NADH. These electrons are transferred through the mitochondrial respiratory chain and ultimately passed to oxygen (O_2_), generating adenosine triphosphate (ATP) through OXPHOS, providing energy to support cell growth and proliferation (Figure 5A). However, in tumors, due to the presence of a HME, hypoxia-inducible factor HIF-1α is activated, leading to significant metabolic reprogramming (Tu et al., 2021). HIF-1α supports tumor cell adaptation to hypoxic conditions by regulating several key enzymes in glucose metabolism (Figure 5B). First, HIF-1α upregulates the expression of *SLC2A1* and *SLC2A3*, which encode glucose transporters GLUT1 and GLUT3, enhancing glucose uptake to meet the high demand for glucose due to the rapid proliferation of tumor cells (Sebestyén et al., 2021). Second, HIF-1α increases the expression of pyruvate dehydrogenase kinase 1 (PDK1), which inhibits the activity of pyruvate dehydrogenase (PDH), thereby preventing pyruvate from being converted into acetyl-CoA and reducing the accumulation of reactive oxygen species (ROS) in the mitochondria, thus protecting cells from hypoxia-induced apoptosis (Korotchkina and Patel, 1995; Korotchkina and Patel, 2001). Additionally, HIF-1α upregulates the expression of lactate dehydrogenase A (LDHA), promoting the conversion of pyruvate to lactate (Semenza et al., 1996; Le et al., 2010; Ma et al., 2014). This conversion shifts tumor cell metabolism from oxidative phosphorylation to glycolysis, generating more energy and intermediate metabolites to support cell proliferation. During glycolysis, 3-phosphoglycerate (3PG) enters the serine synthesis pathway (SSP) to produce serine (Bao and Wong, 2021). Serine then enters the folate cycle, providing nicotinamide adenine dinucleotide phosphate (NADPH) to further neutralize ROS and protect the cells from oxidative stress. These metabolic changes result in tumor cells exhibiting an aerobic glycolysis phenotype (Warburg effect), even under aerobic conditions (Parks et al., 2017). In other words, tumor cells primarily rely on glycolysis to generate energy, supporting their rapid growth, proliferation, and survival. Therefore, HIF-1α plays a key role in the metabolic reprogramming of tumor cells, helping them adapt to the harsh hypoxic environment and sustain their proliferative capacity.

### 6.2 Metabolic shifts in vascular tumors

The role of metabolic reprogramming in IH has gradually gained attention, particularly the alterations in the glycolytic pathway. Chen et al. found that glycolysis-associated molecules, such as GLUT1, hexokinase 2 (HK2), phosphofructokinase 2/6-phosphofructo-2-kinase (PFKFB3), pyruvate kinase M2 (PKM2), and LDHA, were significantly more highly expressed at both the mRNA and protein levels in HemECs compared to human umbilical vein endothelial cells (HUVECs) (Chen et al., 2020). Moreover, HemECs consumed glucose at higher rates. Inhibition of these glycolysis-associated molecules significantly reduced the proliferation, migration, and tube formation abilities of HemECs. Mei et al. found that lncRNA MCM3AP-AS1 promoted the progression of IH by upregulating glycolysis-related genes (such as GLUT1, LDHA, and HK2) through the miR-138-5p/HIF-1α axis (Mei et al., 2021). Yang et al. reported that PFKFB3 was expressed at higher levels in the proliferative phase of IH than in the regressive phase, and its inhibition significantly reduced tumor growth and angiogenesis in IH, highlighting PFKFB3 as a potential new therapeutic target for IH (Yang et al., 2023). Li et al. demonstrated that OTUB1 promoted glycolysis and angiogenesis in HemECs by deubiquitinating TGFBI in a catalytic-independent manner, further emphasizing the role of glycolysis in angiogenesis in IH (Li et al., 2023). Glycolysis plays a central role in the metabolic reprogramming of IH, involving multiple molecules and signaling pathways, and targeting glycolysis-associated molecules provides new potential strategies for the treatment of IH. At the same time, some studies also suggest that lipid metabolism plays an important role in the hypoxia-regulated process in IH. Jiang et al. showed that apolipoprotein A-I binding protein (AIBP) regulates cholesterol metabolism and inhibits hypoxia-induced activation of HIF-1α, thereby reducing angiogenesis in IH, suggesting AIBP as a potential therapeutic target for IH (Jiang et al., 2024).

After KSHV infection, the metabolic activity of infected cells is reprogrammed to favor their persistence, reactivation, and the development of KS (Singh et al., 2022). Singh et al. demonstrated that this metabolic reprogramming includes an increased dependence on glucose, enhanced glucose uptake, and elevated lactate production (Singh et al., 2022; Davis et al., 2023). These changes are partly mediated through HIF-1α, which, when upregulated, leads to the altered expression of key metabolic enzymes, particularly the glucose transporter GLUT1 (Shrestha et al., 2017). Further studies by Ma et al. showed that the metabolic effector of HIF-1, pyruvate kinase M2 (PKM2), one of the isoforms of the kinase involved in the final step reaction of glycolysis, is upregulated in ECs infected with KSHV, thereby mimicking the Warburg effect commonly seen in tumors (Li et al., 2014; Ma et al., 2015). Additionally, PKM2 regulates the KS angiogenic phenotype by acting as a coactivator of HIF-1 and increasing the expression of HIF-1-induced angiogenic factors, such as VEGF (Luo and Semenza, 2012). Inhibition of PKM2 expression not only downregulates the Warburg effect but also significantly suppresses KS-associated angiogenesis, positioning PKM2 as a potential therapeutic target for KS.

## 7 Therapeutic targets in vascular tumors

Based on the mechanisms outlined above, several drugs related to hypoxia-induced pathways have already entered clinical trials (Table 2).

**TABLE 2:** Therapeutic targets in vascular tumors: Clinical trials related to hypoxia-induced pathways.

Molecular target	Trial ID	Drug	Applicable type	Status	Phase	Start Date	Refs
β-receptors	NCT06798363	Propranolol	Ulcerated IH	Not yet recruiting	I/II	2025	/
	NCT04684667	Propranolol	IH	Unknown status	II	2020	/
	2014-005555-80	Propranolol	IH	Completed	III	2015	/
	NCT01512173	Propranolol	IH	Completed	II	2012	/
	2010-023488-16	Propranolol	IH	Completed	III	2011	/
	2011-003144-50	Propranolol	IH	Completed	II	2011	/
	NCT01056341	Propranolol	IH	Completed	II/III	2010	Léauté-Labrèze et al. (2015)
	NCT04651049	Propranolol	IH	Completed	/	2010	/
	NCT01072045	Propranolol	IH	Completed	II	2010	/
	NCT00967226	Propranolol	IH	Completed	II	2009	/
	2009-013262-84	Propranolol	IH	Completed	II/III	2009	/
	NCT01211080	Propranolol	IH	Completed	/	2008	/
	NCT06677853	Timolol	IH	Completed	II/III	2020	/
	NCT04288700	Timolol	IH	Unknown status	IV	2019	/
	NCT02913612	Timolol	IH	Completed	II	2017	Drolet et al. (2020)
	NCT02145884	Timolol	IH	Completed	II	2014	/
	NCT02731287	Timolol	IH	Completed	II	2014	/
	2013-005199-17	Timolol	IH	Completed	III	2014	/
	NCT01434849	Timolol	IH	Completed	I	2012	/
	NCT01685398	Timolol	IH	Completed	III	2012	/
	NCT01408056	Timolol	Ulcerated IH	Withdrawn	II	2011	/
	NCT01147601	Timolol	IH	Completed	I	2010	/
	NCT02505971	Nadolol	IH	Completed	III	2015	/
	NCT01010308	Nadolol	IH	Completed	II	2009	/
	NCT03237637	Atenolol	IH	Unknown status	III	2017	/
	NCT02342275	Atenolol	IH	Completed	III	2013	Ji et al. (2021)
	2011-004145-41	Acebutolol	IH	Ongoing	II	2013	/
	NCT01743885	Acebutolol	IH	Terminated	III	2012	/
	NCT06445166	Propranolol	KS	Not yet recruiting	II	2024	/
	NCT05797662	Propranolol	KS	Not yet recruiting	II	2025	/
	NCT05961761	Propranolol	AS	Recruiting	/	2023	/
	2021-003788-82	Propranolol	AS	Trial now transitioned	II	2021	/
	NCT04518124	Propranolol	AS	Completed	II	2020	Heinhuis et al. (2020)
	2019-002947-41	Propranolol	AS	Completed	II	2019	Heinhuis et al. (2020)
	NCT02732678	Propranolol	AS	Unknown	I/II	2016	/
mTOR	NCT05324384	Sirolimus	KHE	Recruiting	II	2022	/
	NCT04775173	Sirolimus	KHE	Completed	II	2021	/
	NCT04448873	Sirolimus	KHE	Completed	IV	2020	/
	NCT04077515	Sirolimus	KHE	Completed	IV	2019	/
	NCT03188068	Sirolimus	KHE	Completed	II	2017	/
	NCT04406870	Sirolimus	IHHE	Not yet recruiting	IV	2020	/
	NCT01412515	Everolimus	KS	Terminated	II	2008	/
VEGF	NCT01296815	Bevacizumab	KS	Completed	II	2010	/
	NCT00923936	Bevacizumab	KS	Completed	II	2009	/
	NCT00055237	Bevacizumab	KS	Completed	II	2003	/
	NCT01303497	Bevacizumab	AS	Completed	II	2011	Lebellec et al. (2018)
	NCT01055028	Bevacizumab	AS	Terminated	II	2010	/
	NCT00887809	Bevacizumab	AS	Completed	II	2009	/
	NCT00288015	Bevacizumab	AS	Completed	II	2006	/
	2004-004546-41	Bevacizumab	AS	Ongoing	II	2005	/

Compilation of the interventional clinical trials registered at the U.S. National Library of Medicine (https://clinicaltrials.gov, accessed on 8 February 2025) and in the EU Clinical Trials Register (https://www.clinicaltrialsregister.eu, accessed on 8 February 2025). Abbreviation: IH, infantile hemangioma; KHE, kaposiform hemangioendothelioma; IHHE, infantile hepatic hemangioendothelioma; KS, kaposi sarcoma; AS, angiosarcoma.

### 7.1 β-Blockers

Propranolol is a non-selective β1 and β2 adrenergic receptor (ADRB1-2) antagonist initially used for the treatment of cardiovascular diseases. In 2008, Léauté-Labrèze et al. discovered that propranolol could induce apoptosis in capillary ECs, demonstrating its potential as an anti-angiogenic agent (Léauté-Labrèze et al., 2008). Based on this finding, they conducted clinical trials in 11 patients with IH. Following this, a larger randomized controlled trial (NCT01056341, n = 460) demonstrated that administering propranolol at a dosage of 3 mg/kg/day led to complete or near-complete regression in 60% of patients with IH. When started early during the proliferative phase, IH typically regressed within 6 months. After discontinuing the treatment, the recurrence rate was around 10% (Léauté-Labrèze et al., 2015). In addition, propranolol has also shown promising efficacy in the treatment of KHE. A study by Li et al. demonstrated that a dosage of 2 mg/kg/day of propranolol was effective in treating cutaneous KHE, with no severe adverse effects observed and a high level of safety during long-term treatment (Wei et al., 2022). Case reports have also suggested that oral propranolol, when combined with external therapies, can provide adjunctive benefits for KHE management, further supporting its therapeutic potential in this vascular tumor (Dang and Ren, 2024). Due to the exceedingly low incidence of KHE, clinical trials assessing the efficacy of propranolol in this context have not yet been undertaken. Fortunately, clinical investigations exploring the use of propranolol in the treatment of KS and Angiosarcoma AS have been initiated (Heinhuis et al., 2020). At the time of writing this review, these trials remain ongoing.

Currently, the precise mechanisms underlying the use of propranolol in the treatment of vascular tumors remain incompletely elucidated. Potential mechanisms related to hypoxia induction include a significant reduction in VEGFA levels in the blood of IH patients treated with propranolol, which may occur through the downregulation of HIFs(Lorusso et al., 2022; Makkeyah et al., 2022). Additionally, propranolol may exert its effects through the regulation of metabolic reprogramming. Existing studies have shown that propranolol reduces the production of pyruvate and lactate during glycolysis by downregulating HK2 activity, lowering glucose-6-phosphate levels, without affecting fructose-1,6-bisphosphate concentration, thus altering the energy supply in tumor lesions (Xiang et al., 2024; Leonard, 1972; Kang et al., 2014). In summary, propranolol, by modulating hypoxic responses and metabolic reprogramming, may play a significant role in the treatment of vascular tumors.

In addition to propranolol, β-blockers currently undergoing clinical trials include Timolol, Nadolol, Atenolol, and Acebutolol. In a clinical trial involving 76 children (NCT02913612), the topical application of 0.5% Timolol showed significant efficacy, but it was only effective for small, thin IH (Drolet et al., 2020). As the thickness of the IH increased, the treatment effect became less pronounced. Ji et al. conducted a comparison of propranolol and Atenolol for the treatment of IH (NCT02342275) (Ji et al., 2021). The efficacy of both treatments was similar, but adverse reactions were less common in the Atenolol group. These novel β-blockers may become a first-line treatment for IH in the future, but their mechanisms of action have not been thoroughly investigated.

### 7.2 Sirolimus

HIF-1α is a downstream target of the mTOR signaling pathway, where mTOR signaling can stabilize and enhance the transcriptional activity of HIF-1α (Wang et al., 2024). Sirolimus can target mTOR to inhibit HIF-1α and its downstream molecules, such as VEGF. In vascular tumors, sirolimus is widely used in the treatment of KHE (Qiu et al., 2025). Retrospective studies have shown that, regardless of the presence or absence of KMP, sirolimus achieves an overall efficacy rate of over 90% in patients with KHE (Wang Z. et al., 2019; Borst et al., 2024). Multiple prospective studies and randomized controlled trials (RCTs) have also confirmed this (Adams et al., 2016; Freixo et al., 2020; Ji et al., 2022). However, it remains uncertain whether sirolimus exerts its therapeutic effect through the HIFs pathway. Moreover, several clinical trials have started exploring the use of sirolimus for the treatment of Infantile Hepatic Hemangioendothelioma (IHHE) and KS.

### 7.3 Bevacizumab

Bevacizumab (Avastin®, F. Hoffmann-La Roche AG, Switzerland) is a human monoclonal antibody targeting VEGFA and represents the first approved anti-angiogenic agent (Garcia et al., 2020). Bevacizumab exerts its therapeutic effect by binding to VEGFA, thereby preventing its interaction with the VEGFR2 receptor. This mechanism inhibits the activation of the VEGF signaling pathway, blocks the formation of new tumor blood vessels, reduces the tumor’s blood supply, and consequently suppresses tumor growth and metastasis. Currently, Bevacizumab has been approved for the treatment of various solid tumors, including colorectal cancer, non-small cell lung cancer, ovarian cancer, and renal cell carcinoma (Des Guetz et al., 2006; Seto et al., 2006; Paley et al., 1997; Gao and McDermott, 2018).

In addition to its widespread application in common solid tumors, Bevacizumab has also been investigated in clinical trials for the treatment of vascular tumors, particularly KS and AS. For example, in a trial (NCT00923936) evaluating the combination of liposomal doxorubicin and Bevacizumab for the treatment of advanced KS in adult patients, although no cases of complete remission were observed, relatively favorable outcomes were achieved in HIV-associated KS patients. Further research, such as the trial in NCT00055237, assessed the efficacy of Bevacizumab in both HIV-positive and HIV-negative KS patients. In HIV-positive patients, 31% (range: 11%–58.7%) of patients showed either complete or partial remission. However, in clinical trials evaluating the treatment of AS, Bevacizumab has not demonstrated significant efficacy. A study by Lebellec et al. (NCT01303497) assessed the combination of Bevacizumab and paclitaxel for the treatment of AS (Lebellec et al., 2018). The results indicated that although Bevacizumab was able to significantly reduce tumor burden in the short term, it did not lead to a significant improvement in progression-free survival (PFS). This suggests that the long-term efficacy of Bevacizumab in angiosarcoma remains limited.

## 8 Summary

In recent decades, considerable advancements have been made in elucidating the signaling pathways mediated by hypoxia. This has also been the case in the study of vascular tumors, where hypoxia represents a pivotal characteristic of the tumor microenvironment. Hypoxia induces the stabilization of HIF-1α, which subsequently activates key pro-angiogenic pathways, including the upregulation of VEGF. This leads to the formation of aberrant and dysfunctional blood vessels, which further exacerbate the hypoxic conditions within the tumor. In parallel, hypoxia triggers a metabolic shift toward enhanced glycolysis, even in the presence of oxygen, a hallmark of the Warburg effect. This metabolic adaptation not only supports tumor cell survival and proliferation but also contributes to the invasive potential of tumors. Targeting the hypoxia-driven processes of angiogenesis and metabolic reprogramming holds significant promise for the treatment of vascular tumors. However, these strategies remain primarily in the preclinical stage, and further research is required to assess their clinical feasibility and therapeutic efficacy.

## References

[B1] Abedalthagafi M. Rushing E. J. Auerbach A. Desouki M. M. Marwaha J. Wang Z. (2010). Sporadic cutaneous angiosarcomas generally lack hypoxia-inducible factor 1*α*: a histologic and immunohistochemical study of 45 cases. Annals Diagnostic Pathology 14, 15–22. 10.1016/j.anndiagpath.2009.09.005 20123452

[B2] Adams D. M. Trenor C. C. Hammill A. M. Vinks A. A. Patel M. N. Chaudry G. (2016). Efficacy and safety of sirolimus in the treatment of complicated vascular anomalies. Pediatrics 137, e20153257. 10.1542/peds.2015-3257 26783326 PMC4732362

[B3] Albores-Saavedra J. Schwartz A. M. Henson D. E. Kostun L. Hart A. Angeles-Albores D. (2011). Cutaneous angiosarcoma. Analysis of 434 cases from the surveillance, epidemiology, and end results program, 1973-2007. Ann. Diagn Pathol. 15, 93–97. 10.1016/j.anndiagpath.2010.07.012 21190880

[B4] Al-Salam S. Balalaa N. Faour I. Akhter S. Alashari M. (2012). HIF-1α, VEGF and WT-1 are protagonists in bilateral primary angiosarcoma of breast: a case report and review of literature. Int. J. Clin. Exp. Pathol. 5, 247–253. 22558480 PMC3341685

[B5] Amir J. Metzker A. Krikler R. Reisner S. H. (1986). Strawberry hemangioma in preterm infants. Pediatr. Dermatol 3, 331–332. 10.1111/j.1525-1470.1986.tb00535.x 3774653

[B6] An R. Men X.-J. Ni X.-H. Wang W.-T. Wang C.-L. (2024). Angiosarcoma of the breast: a review. Heliyon 10, e24413. 10.1016/j.heliyon.2024.e24413 38318005 PMC10839862

[B7] Aneja K. K. Yuan Y. (2017). Reactivation and lytic replication of Kaposi’s sarcoma-associated herpesvirus: an update. Front. Microbiol. 8, 613. 10.3389/fmicb.2017.00613 28473805 PMC5397509

[B8] Azzariti A. Porcelli L. Mangia A. Saponaro C. Quatrale A. E. Popescu O. S. (2014). Irradiation-induced angiosarcoma and anti-angiogenic therapy: a therapeutic hope? Exp. Cell Res. 321, 240–247. 10.1016/j.yexcr.2013.12.018 24384475

[B9] Baluk P. Hashizume H. McDonald D. M. (2005). Cellular abnormalities of blood vessels as targets in cancer. Curr. Opin. Genet. Dev. 15, 102–111. 10.1016/j.gde.2004.12.005 15661540

[B10] Bao M. H.-R. Wong C. C.-L. (2021). Hypoxia, metabolic reprogramming, and drug resistance in liver cancer. Cells 10, 1715. 10.3390/cells10071715 34359884 PMC8304710

[B11] Bao X. Zhang J. Huang G. Yan J. Xu C. Dou Z. (2021). The crosstalk between HIFs and mitochondrial dysfunctions in cancer development. Cell Death Dis. 12, 215. 10.1038/s41419-021-03505-1 33637686 PMC7910460

[B12] Baselga E. Roe E. Coulie J. Muñoz F. Z. Boon L. M. McCuaig C. (2016). Risk factors for degree and type of sequelae after involution of untreated hemangiomas of infancy. JAMA Dermatol 152, 1239–1243. 10.1001/jamadermatol.2016.2905 27540637

[B13] Bauland C. G. Lüning T. H. Smit J. M. Zeebregts C. J. Spauwen P. H. M. (2011). Untreated hemangiomas: growth pattern and residual lesions. Plast. Reconstr. Surg. 127, 1643–1648. 10.1097/PRS.0b013e318208d2ac 21460670

[B14] Bi S. Zhong A. Yin X. Li J. Cen Y. Chen J. (2022). Management of cutaneous angiosarcoma: an update review. Curr. Treat. Options Oncol. 23, 137–154. 10.1007/s11864-021-00933-1 35182299

[B15] Borsi E. Terragna C. Brioli A. Tacchetti P. Martello M. Cavo M. (2015). Therapeutic targeting of hypoxia and hypoxia-inducible factor 1 alpha in multiple myeloma. Transl. Res. 165, 641–650. 10.1016/j.trsl.2014.12.001 25553605

[B16] Borst A. J. Eng W. Griffin M. Ricci K. W. Engel E. Adams D. M. (2024). Treatment practices and response in kaposiform hemangioendothelioma: a multicenter cohort study. Pediatr. Blood Cancer 71, e30779. 10.1002/pbc.30779 38073018

[B17] Bos R. van der Groep P. Greijer A. E. Shvarts A. Meijer S. Pinedo H. M. (2003). Levels of hypoxia-inducible factor-1alpha independently predict prognosis in patients with lymph node negative breast carcinoma. Cancer 97, 1573–1581. 10.1002/cncr.11246 12627523

[B18] Cai Q. Cai S. Zhu C. Verma S. C. Choi J.-Y. Robertson E. S. (2013). A unique SUMO-2-interacting motif within LANA is essential for KSHV latency. PLoS Pathog. 9, e1003750. 10.1371/journal.ppat.1003750 24278015 PMC3836728

[B19] Cai Q. Lan K. Verma S. C. Si H. Lin D. Robertson E. S. (2006a). Kaposi’s sarcoma-associated herpesvirus latent protein LANA interacts with HIF-1 alpha to upregulate RTA expression during hypoxia: latency control under low oxygen conditions. J. Virol. 80, 7965–7975. 10.1128/JVI.00689-06 16873253 PMC1563785

[B20] Cai Q.-L. Knight J. S. Verma S. C. Zald P. Robertson E. S. (2006b). EC5S ubiquitin complex is recruited by KSHV latent antigen LANA for degradation of the VHL and p53 tumor suppressors. PLoS Pathog. 2, e116. 10.1371/journal.ppat.0020116 17069461 PMC1626105

[B21] Cao J. Wang J. He C. Fang M. (2019). Angiosarcoma: a review of diagnosis and current treatment. Am. J. Cancer Res. 9, 2303–2313. 31815036 PMC6895451

[B22] Catrina S.-B. Botusan I. R. Rantanen A. Catrina A. I. Pyakurel P. Savu O. (2006). Hypoxia-inducible factor-1alpha and hypoxia-inducible factor-2alpha are expressed in kaposi sarcoma and modulated by insulin-like growth factor-I. Clin. Cancer Res. 12, 4506–4514. 10.1158/1078-0432.CCR-05-2473 16899596

[B23] Cesarman E. Damania B. Krown S. E. Martin J. Bower M. Whitby D. (2019). Kaposi sarcoma. Nat. Rev. Dis. Prim. 5, 9. 10.1038/s41572-019-0060-9 30705286 PMC6685213

[B24] Chang Y. Cesarman E. Pessin M. S. Lee F. Culpepper J. Knowles D. M. (1994). Identification of herpesvirus-like DNA sequences in AIDS-associated Kaposi’s sarcoma. Science 266, 1865–1869. 10.1126/science.7997879 7997879

[B25] Chaudhary P. Bhadana U. Singh R. a. K. Ahuja A. (2015). Primary hepatic angiosarcoma. Eur. J. Surg. Oncol. 41, 1137–1143. 10.1016/j.ejso.2015.04.022 26008857

[B26] Chen C.-Y. Chen J. He L. Stiles B. L. (2018). PTEN: tumor suppressor and metabolic regulator. Front. Endocrinol. 9, 338. 10.3389/fendo.2018.00338 PMC6046409 30038596

[B27] Chen J. Wu D. Dong Z. Chen A. Liu S. (2020). The expression and role of glycolysis-associated molecules in infantile hemangioma. Life Sci. 259, 118215. 10.1016/j.lfs.2020.118215 32768579

[B28] Chen Z. Han F. Du Y. Shi H. Zhou W. (2023). Hypoxic microenvironment in cancer: molecular mechanisms and therapeutic interventions. Signal Transduct. Target Ther. 8, 70. 10.1038/s41392-023-01332-8 36797231 PMC9935926

[B29] Chen Z.-Y. Wang Q.-N. Zhu Y.-H. Zhou L.-Y. Xu T. He Z.-Y. (2019). Progress in the treatment of infantile hemangioma. Ann. Transl. Med. 7, 692. 10.21037/atm.2019.10.47 31930093 PMC6944559

[B30] Choi Y. K. (2017). A positive circuit of VEGF increases Glut-1 expression by increasing HIF-1α gene expression in human retinal endothelial cells. Arch. Pharm. Res. 40, 1433–1442. 10.1007/s12272-017-0971-5 29022192

[B31] Codo A. C. Davanzo G. G. Monteiro L. de B. de Souza G. F. Muraro S. P. Virgilio-da-Silva J. V. (2020). Elevated glucose levels favor SARS-CoV-2 infection and monocyte response through a HIF-1α/Glycolysis-Dependent Axis. Cell Metab. 32, 437–446.e5. 10.1016/j.cmet.2020.07.007 32697943 PMC7367032

[B32] Cohen O. G. Florez-Pollack S. Finn L. S. Larijani M. Jen M. Treat J. (2022). Multifocal kaposiform hemangioendothelioma in a newborn with confirmatory histopathology. Pediatrics 150, e2022056293. 10.1542/peds.2022-056293 36193691

[B33] Colonna V. Resta L. Napoli A. Bonifazi E. (2010). Placental hypoxia and neonatal haemangioma: clinical and histological observations. Br. J. Dermatology 162, 208–209. 10.1111/j.1365-2133.2009.09493.x 19863512

[B34] Couto R. A. Maclellan R. A. Zurakowski D. Greene A. K. (2012). Infantile hemangioma: clinical assessment of the involuting phase and implications for management. Plast. Reconstr. Surg. 130, 619–624. 10.1097/PRS.0b013e31825dc129 22575857

[B35] Crawford Y. Kasman I. Yu L. Zhong C. Wu X. Modrusan Z. (2009). PDGF-C mediates the angiogenic and tumorigenic properties of fibroblasts associated with tumors refractory to anti-VEGF treatment. Cancer Cell 15, 21–34. 10.1016/j.ccr.2008.12.004 19111878

[B36] Croteau S. E. Liang M. G. Kozakewich H. P. Alomari A. I. Fishman S. J. Mulliken J. B. (2013). Kaposiform hemangioendothelioma: atypical features and risks of Kasabach-Merritt phenomenon in 107 referrals. J. Pediatr. 162, 142–147. 10.1016/j.jpeds.2012.06.044 22871490 PMC3494787

[B37] Curtiss P. Strazzulla L. C. Friedman-Kien A. E. (2016). An update on Kaposi’s sarcoma: epidemiology, pathogenesis and treatment. Dermatol Ther. (Heidelb) 6, 465–470. 10.1007/s13555-016-0152-3 27804093 PMC5120640

[B38] Dachs G. U. Dougherty G. J. Stratford I. J. Chaplin D. J. (1997). Targeting gene therapy to cancer: a review. Oncol. Res. 9, 313–325. 9406237

[B39] Dales J.-P. Garcia S. Meunier-Carpentier S. Andrac-Meyer L. Haddad O. Lavaut M.-N. (2005). Overexpression of hypoxia-inducible factor HIF-1alpha predicts early relapse in breast cancer: retrospective study in a series of 745 patients. Int. J. Cancer 116, 734–739. 10.1002/ijc.20984 15849727

[B40] Dang N. Ren Y. (2024). A case of superficial kaposiform hemangioendothelioma treated with oral propranolol combined with topical sirolimus. Vasc. Health Risk Manag. 20, 251–254. 10.2147/VHRM.S461505 38883398 PMC11180431

[B41] Davis D. A. Shrestha P. Yarchoan R. (2023). Hypoxia and hypoxia-inducible factors in Kaposi sarcoma-associated herpesvirus infection and disease pathogenesis. J. Med. Virol. 95, e29071. 10.1002/jmv.29071 37665216 PMC10502919

[B42] Deeb G. Vaughan M. M. McInnis I. Ford L. A. Sait S. N. J. Starostik P. (2011). Hypoxia-inducible factor-1α protein expression is associated with poor survival in normal karyotype adult acute myeloid leukemia. Leuk. Res. 35, 579–584. 10.1016/j.leukres.2010.10.020 21176961

[B43] de Jong S. Itinteang T. Withers A. H. J. Davis P. F. Tan S. T. (2016). Does hypoxia play a role in infantile hemangioma? Arch. Dermatol Res. 308, 219–227. 10.1007/s00403-016-1635-x 26940670

[B44] Dengler V. L. Galbraith M. Espinosa J. M. (2014). Transcriptional regulation by hypoxia inducible factors. Crit. Rev. Biochem. Mol. Biol. 49, 1–15. 10.3109/10409238.2013.838205 24099156 PMC4342852

[B45] Des Guetz G. Uzzan B. Nicolas P. Cucherat M. Morere J.-F. Benamouzig R. (2006). Microvessel density and VEGF expression are prognostic factors in colorectal cancer. Meta-analysis of the literature. Br. J. Cancer 94, 1823–1832. 10.1038/sj.bjc.6603176 16773076 PMC2361355

[B46] Ding Y. Zhang J.-Z. Yu S.-R. Xiang F. Kang X.-J. (2020). Risk factors for infantile hemangioma: a meta-analysis. World J. Pediatr. 16, 377–384. 10.1007/s12519-019-00327-2 31853885

[B47] Disharoon M. Kozlowski K. F. Kaniowski J. M. (2017). Case 242: radiation-induced angiosarcoma. Radiology 283, 909–916. 10.1148/radiol.2017150456 28514220

[B48] Drolet B. A. Boakye-Agyeman F. Harper B. Holland K. Lewandowski A. Stefanko N. (2020). Systemic timolol exposure following topical application to infantile hemangiomas. J. Am. Acad. Dermatology 82, 733–736. 10.1016/j.jaad.2019.02.029 30790601

[B49] Drolet B. A. Frieden I. J. (2010). Characteristics of infantile hemangiomas as clues to pathogenesis: does hypoxia connect the dots? Archives Dermatology 146, 1295–1299. 10.1001/archdermatol.2010.1295 21079070

[B50] Drolet B. A. Trenor C. C. Brandão L. R. Chiu Y. E. Chun R. H. Dasgupta R. (2013). Consensus-derived practice standards plan for complicated Kaposiform hemangioendothelioma. J. Pediatr. 163, 285–291. 10.1016/j.jpeds.2013.03.080 23796341

[B51] Dufresne A. Meeus P. Sunyach M.-P. (2023). Treatment of radiation-associated angiosarcoma. Curr. Opin. Oncol. 35, 296–300. 10.1097/CCO.0000000000000958 37222197

[B52] Eisenstein K. A. (2023). Infantile hemangiomas: a review and future opportunities. Mo Med. 120, 49–52. 36860594 PMC9970331

[B53] Elhanani O. Ben-Uri R. Keren L. (2023). Spatial profiling technologies illuminate the tumor microenvironment. Cancer Cell 41, 404–420. 10.1016/j.ccell.2023.01.010 36800999

[B54] El-Mallawany N. K. Kamiyango W. Slone J. S. Villiera J. Kovarik C. L. Cox C. M. (2016). Clinical factors associated with long-term complete remission versus poor response to chemotherapy in HIV-infected children and adolescents with kaposi sarcoma receiving bleomycin and vincristine: a retrospective observational study. PLoS One 11, e0153335. 10.1371/journal.pone.0153335 27082863 PMC4833299

[B55] Eltzschig H. K. Carmeliet P. (2011). Hypoxia and inflammation. N. Engl. J. Med. 364, 656–665. 10.1056/NEJMra0910283 21323543 PMC3930928

[B56] Evens A. M. Sehn L. H. Farinha P. Nelson B. P. Raji A. Lu Y. (2010). Hypoxia-inducible factor-1α expression predicts superior survival in patients with diffuse large B-cell lymphoma treated with R-CHOP. J. Clin. Oncol. 28, 1017–1024. 10.1200/JCO.2009.24.1893 20048181 PMC2834428

[B57] Fayette J. Martin E. Piperno-Neumann S. Le Cesne A. Robert C. Bonvalot S. (2007). Angiosarcomas, a heterogeneous group of sarcomas with specific behavior depending on primary site: a retrospective study of 161 cases. Ann. Oncol. 18, 2030–2036. 10.1093/annonc/mdm381 17974557

[B58] Fernández Y. Bernabeu-Wittel M. García-Morillo J. S. (2009). Kaposiform hemangioendothelioma. Eur. J. Intern Med. 20, 106–113. 10.1016/j.ejim.2008.06.008 19327597

[B59] Firth J. D. Ebert B. L. Pugh C. W. Ratcliffe P. J. (1994). Oxygen-regulated control elements in the phosphoglycerate kinase 1 and lactate dehydrogenase A genes: similarities with the erythropoietin 3’ enhancer. Proc. Natl. Acad. Sci. U. S. A. 91, 6496–6500. 10.1073/pnas.91.14.6496 8022811 PMC44229

[B60] Folpe A. L. (2024). Vascular tumors of intermediate malignancy: an update. Hum. Pathol. 147, 114–128. 10.1016/j.humpath.2024.01.014 38360216

[B61] Forbess Smith C. J. Friedlander S. F. Guma M. Kavanaugh A. Chambers C. D. (2017). Infantile hemangiomas: an updated review on risk factors, pathogenesis, and treatment. Birth Defects Res. 109, 809–815. 10.1002/bdr2.1023 28402073 PMC5839165

[B62] Forde K. M. Glover M. T. Chong W. K. Kinsler V. A. (2017). Segmental hemangioma of the head and neck: high prevalence of PHACE syndrome. J. Am. Acad. Dermatol 76, 356–358. 10.1016/j.jaad.2016.06.058 28089002

[B63] Freedman S. J. Sun Z.-Y. J. Poy F. Kung A. L. Livingston D. M. Wagner G. (2002). Structural basis for recruitment of CBP/p300 by hypoxia-inducible factor-1 alpha. Proc. Natl. Acad. Sci. U. S. A. 99, 5367–5372. 10.1073/pnas.082117899 11959990 PMC122775

[B64] Freixo C. Ferreira V. Martins J. Almeida R. Caldeira D. Rosa M. (2020). Efficacy and safety of sirolimus in the treatment of vascular anomalies: a systematic review. J. Vasc. Surg. 71, 318–327. 10.1016/j.jvs.2019.06.217 31676179

[B65] Frolova O. Samudio I. Benito J. M. Jacamo R. Kornblau S. M. Markovic A. (2012). Regulation of HIF-1α signaling and chemoresistance in acute lymphocytic leukemia under hypoxic conditions of the bone marrow microenvironment. Cancer Biol. Ther. 13, 858–870. 10.4161/cbt.20838 22785211 PMC3414410

[B66] Fury M. G. Antonescu C. R. Van Zee K. J. Brennan M. F. Maki R. G. (2005). A 14-year retrospective review of angiosarcoma: clinical characteristics, prognostic factors, and treatment outcomes with surgery and chemotherapy. Cancer J. 11, 241–247. 10.1097/00130404-200505000-00011 16053668

[B67] Gao X. McDermott D. F. (2018). Combinations of bevacizumab with immune checkpoint inhibitors in renal cell carcinoma. Cancer J. 24, 171–179. 10.1097/PPO.0000000000000323 30119080

[B68] Garcia J. Hurwitz H. I. Sandler A. B. Miles D. Coleman R. L. Deurloo R. (2020). Bevacizumab (Avastin®) in cancer treatment: a review of 15 years of clinical experience and future outlook. Cancer Treat. Rev. 86, 102017. 10.1016/j.ctrv.2020.102017 32335505

[B69] Glaunsinger B. Ganem D. (2004). Highly selective escape from KSHV-mediated host mRNA shutoff and its implications for viral pathogenesis. J. Exp. Med. 200, 391–398. 10.1084/jem.20031881 15289507 PMC2211977

[B70] Gomez-Acevedo H. Dai Y. Strub G. Shawber C. Wu J. K. Richter G. T. (2020). Identification of putative biomarkers for Infantile Hemangiomas and Propranolol treatment via data integration. Sci. Rep. 10, 3261. 10.1038/s41598-020-60025-2 32094357 PMC7039967

[B71] Gottlieb G. J. Ragaz A. Vogel J. V. Friedman-Kien A. Rywlin A. M. Weiner E. A. (1981). A preliminary communication on extensively disseminated Kaposi’s sarcoma in young homosexual men. Am. J. Dermatopathol. 3, 111–114. 10.1097/00000372-198100320-00002 7270808

[B72] Greenberger S. Bischoff J. (2011). Infantile hemangioma-mechanism(s) of drug action on a vascular tumor. Cold Spring Harb. Perspect. Med. 1, a006460. 10.1101/cshperspect.a006460 22229118 PMC3234458

[B73] Gruber G. Greiner R. H. Hlushchuk R. Aebersold D. M. Altermatt H. J. Berclaz G. (2004). Hypoxia-inducible factor 1 alpha in high-risk breast cancer: an independent prognostic parameter? Breast Cancer Res. 6, R191–R198. 10.1186/bcr775 15084243 PMC400672

[B74] Grulich A. E. Vajdic C. M. (2015). The epidemiology of cancers in human immunodeficiency virus infection and after organ transplantation. Semin. Oncol. 42, 247–257. 10.1053/j.seminoncol.2014.12.029 25843729

[B75] Günther H. Vaupel P. Metzger H. Thews G. (1972). Stationäre Verteilung der O2-Drucke im Tumorgewebe (DS-Carcinosarkom) I. Messungen *in vivo* unter Verwendung von Gold-Mikroelektroden. Z. Krebsforsch. 77, 26–39. 10.1007/BF00284351 4259984

[B76] Haase V. H. (2013). Regulation of erythropoiesis by hypoxia-inducible factors. Blood Rev. 27, 41–53. 10.1016/j.blre.2012.12.003 23291219 PMC3731139

[B77] Haggstrom A. N. Drolet B. A. Baselga E. Chamlin S. L. Garzon M. C. Horii K. A. (2007). Prospective study of infantile hemangiomas: demographic, prenatal, and perinatal characteristics. J. Pediatr. 150, 291–294. 10.1016/j.jpeds.2006.12.003 17307549

[B78] Haque M. Wang V. Davis D. A. Zheng Z.-M. Yarchoan R. (2006). Genetic organization and hypoxic activation of the Kaposi’s sarcoma-associated herpesvirus ORF34-37 gene cluster. J. Virol. 80, 7037–7051. 10.1128/JVI.00553-06 16809309 PMC1489055

[B79] Heinhuis K. M. Ijzerman N. S. Koenen A. M. van der Graaf W. T. A. Haas R. L. Beijnen J. H. (2020). PropAngio study protocol: a neoadjuvant trial on the efficacy of propranolol monotherapy in cutaneous angiosarcoma-a proof of principle study. BMJ Open 10, e039449. 10.1136/bmjopen-2020-039449 PMC7485254 32912994

[B80] Helczynska K. Larsson A.-M. Holmquist Mengelbier L. Bridges E. Fredlund E. Borgquist S. (2008). Hypoxia-inducible factor-2alpha correlates to distant recurrence and poor outcome in invasive breast cancer. Cancer Res. 68, 9212–9220. 10.1158/0008-5472.CAN-08-1135 19010893

[B81] Herbert A. Ng H. Jessup W. Kockx M. Cartland S. Thomas S. R. (2011). Hypoxia regulates the production and activity of glucose transporter‐1 and indoleamine 2,3‐dioxygenase in monocyte‐derived endothelial‐like cells: possible relevance to infantile haemangioma pathogenesis. Br. J. Dermatology 164, 308–315. 10.1111/j.1365-2133.2010.10086.x 21039406

[B82] Höckel M. Knoop C. Schlenger K. Vorndran B. Bauβnann E. Mitze M. (1993). Intratumoral pO2 predicts survival in advanced cancer of the uterine cervix. Radiotherapy Oncol. 26, 45–50. 10.1016/0167-8140(93)90025-4 8438086

[B83] Höckel M. Schlenger K. Aral B. Mitze M. Schäffer U. Vaupel P. (1996). Association between tumor hypoxia and malignant progression in advanced cancer of the uterine Cervix1. Cancer Res. 56, 4509–4515. 8813149

[B84] Höckel M. Schlenger K. Knoop C. Vaupel P. (1991). Oxygenation of carcinomas of the uterine cervix: evaluation by computerized O2 tension measurements. Cancer Res. 51, 6098–6102. 1933873

[B85] Hon W.-C. Wilson M. I. Harlos K. Claridge T. D. W. Schofield C. J. Pugh C. W. (2002). Structural basis for the recognition of hydroxyproline in HIF-1 alpha by pVHL. Nature 417, 975–978. 10.1038/nature00767 12050673

[B86] Hosaka K. Yang Y. Seki T. Du Q. Jing X. He X. (2020). Therapeutic paradigm of dual targeting VEGF and PDGF for effectively treating FGF-2 off-target tumors. Nat. Commun. 11, 3704. 10.1038/s41467-020-17525-6 32709869 PMC7382445

[B87] Hoshina D. Abe R. Yoshioka N. Saito N. Hata H. Fujita Y. (2013). Establishment of a novel experimental model of human angiosarcoma and a VEGF-targeting therapeutic experiment. J. Dermatological Sci. 70, 116–122. 10.1016/j.jdermsci.2013.02.008 23522954

[B88] Hsieh J. J. Purdue M. P. Signoretti S. Swanton C. Albiges L. Schmidinger M. (2017). Renal cell carcinoma. Nat. Rev. Dis. Prim. 3, 17009. 10.1038/nrdp.2017.9 28276433 PMC5936048

[B89] Iliopoulos O. Levy A. P. Jiang C. Kaelin W. G. Goldberg M. A. (1996). Negative regulation of hypoxia-inducible genes by the von Hippel-Lindau protein. Proc. Natl. Acad. Sci. U. S. A. 93, 10595–10599. 10.1073/pnas.93.20.10595 8855223 PMC38198

[B90] Ivan M. Kondo K. Yang H. Kim W. Valiando J. Ohh M. (2001). HIFalpha targeted for VHL-mediated destruction by proline hydroxylation: implications for O2 sensing. Science 292, 464–468. 10.1126/science.1059817 11292862

[B91] Jaakkola P. Mole D. R. Tian Y. M. Wilson M. I. Gielbert J. Gaskell S. J. (2001). Targeting of HIF-alpha to the von Hippel-Lindau ubiquitylation complex by O2-regulated prolyl hydroxylation. Science 292, 468–472. 10.1126/science.1059796 11292861

[B92] Janmohamed S. R. Brinkhuizen T. den Hollander J. C. Madern G. C. de Laat P. C. van Steensel M. A. (2015). Support for the hypoxia theory in the pathogenesis of infantile haemangioma. Clin. Exp. Dermatol 40, 431–437. 10.1111/ced.12557 25511669

[B93] Jaworska M. Szczudło J. Pietrzyk A. Shah J. Trojan S. E. Ostrowska B. (2023). The Warburg effect: a score for many instruments in the concert of cancer and cancer niche cells. Pharmacol. Rep. 75, 876–890. 10.1007/s43440-023-00504-1 37332080 PMC10374743

[B94] Jham B. C. Ma T. Hu J. Chaisuparat R. Friedman E. R. Pandolfi P. P. (2011). Amplification of the angiogenic signal through the activation of the TSC/mTOR/HIF Axis by the KSHV vGPCR in Kaposi’s sarcoma. PLOS ONE 6, e19103. 10.1371/journal.pone.0019103 21559457 PMC3084756

[B95] Ji X. Wang H. Zhu J. Tang Y. Zhou Y. Zhu L. (2013). Correlation of Nrf2 and HIF-1α in glioblastoma and their relationships to clinicopathologic features and survival. Neurol. Res. 35, 1044–1050. 10.1179/1743132813Y.0000000251 24070025

[B96] Ji Y. Chen S. Yang K. Xia C. Li L. (2020). Kaposiform hemangioendothelioma: current knowledge and future perspectives. Orphanet J. Rare Dis. 15, 39. 10.1186/s13023-020-1320-1 32014025 PMC6998257

[B97] Ji Y. Chen S. Yang K. Zhang X. Zhou J. Li L. (2021). Efficacy and safety of propranolol vs Atenolol in infants with problematic infantile hemangiomas: a randomized clinical trial. JAMA Otolaryngol. Head. Neck Surg. 147, 599–607. 10.1001/jamaoto.2021.0454 33856430 PMC8050788

[B98] Ji Y. Chen S. Zhou J. Yang K. Zhang X. Xiang B. (2022). Sirolimus plus prednisolone vs sirolimus monotherapy for kaposiform hemangioendothelioma: a randomized clinical trial. Blood 139, 1619–1630. 10.1182/blood.2021014027 35030255

[B99] Ji Y. Yang K. Peng S. Chen S. Xiang B. Xu Z. (2018). Kaposiform haemangioendothelioma: clinical features, complications and risk factors for Kasabach-Merritt phenomenon. Br. J. Dermatol 179, 457–463. 10.1111/bjd.16601 29603128 PMC11032113

[B100] Jiang X. Wang J. Deng X. Xiong F. Zhang S. Gong Z. (2020). The role of microenvironment in tumor angiogenesis. J. Exp. Clin. Cancer Res. 39, 204. 10.1186/s13046-020-01709-5 32993787 PMC7526376

[B101] Jiang Y. Li X. Liu Q. Lei G. Wu C. Chen L. (2024). Apolipoprotein A-I binding protein inhibits the formation of infantile hemangioma through cholesterol-regulated hypoxia-inducible factor 1α activation. J. Invest Dermatol 144, 645–658.e7. 10.1016/j.jid.2023.07.030 37832842

[B102] Jin M.-S. Lee H. Park I. A. Chung Y. R. Im S.-A. Lee K.-H. (2016). Overexpression of HIF1α and CAXI predicts poor outcome in early-stage triple negative breast cancer. Virchows Arch. 469, 183–190. 10.1007/s00428-016-1953-6 27184798

[B103] Johnson E. F. Davis D. M. Tollefson M. M. Fritchie K. Gibson L. E. (2018). Vascular tumors in infants: case report and review of clinical, histopathologic, and immunohistochemical characteristics of infantile hemangioma, pyogenic granuloma, noninvoluting congenital hemangioma, tufted angioma, and kaposiform hemangioendothelioma. Am. J. Dermatopathol. 40, 231–239. 10.1097/DAD.0000000000000983 29561329

[B105] Jung J. E. Kim H. S. Lee C. S. Shin Y.-J. Kim Y.-N. Kang G.-H. (2008). STAT3 inhibits the degradation of HIF-1alpha by pVHL-mediated ubiquitination. Exp. Mol. Med. 40, 479–485. 10.3858/emm.2008.40.5.479 18985005 PMC2679355

[B106] Kang F. Ma W. Ma X. Shao Y. Yang W. Chen X. (2014). Propranolol inhibits glucose metabolism and 18F-fdg uptake of breast cancer through posttranscriptional downregulation of hexokinase-2. J. Nucl. Med. 55, 439–445. 10.2967/jnumed.113.121327 24504055 PMC4564063

[B107] Kaposi M. (1872). Idiopathisches multiples Pigmentsarkom der Haut. Arch. F. Dermat. 4, 265–273. 10.1007/BF01830024

[B108] Kasabach H. H. Merritt K. K. (1940). Capillary hemangioma with extensive purpura: report of a case. Am. J. Dis. Child. 59, 1063–1070. 10.1001/archpedi.1940.01990160135009

[B109] Kleinman M. E. Greives M. R. Churgin S. S. Blechman K. M. Chang E. I. Ceradini D. J. (2007). Hypoxia-induced mediators of stem/progenitor cell trafficking are increased in children with hemangioma. Arterioscler. Thromb. Vasc. Biol. 27, 2664–2670. 10.1161/ATVBAHA.107.150284 17872454

[B110] Konieczny P. Xing Y. Sidhu I. Subudhi I. Mansfield K. P. Hsieh B. (2022). Interleukin-17 governs hypoxic adaptation of injured epithelium. Science 377, eabg9302. 10.1126/science.abg9302 35709248 PMC9753231

[B111] Korbecki J. Simińska D. Gąssowska-Dobrowolska M. Listos J. Gutowska I. Chlubek D. (2021). Chronic and cycling hypoxia: drivers of cancer chronic inflammation through HIF-1 and NF-κB activation: a review of the molecular mechanisms. Int. J. Mol. Sci. 22, 10701. 10.3390/ijms221910701 34639040 PMC8509318

[B112] Korotchkina L. G. Patel M. S. (1995). Mutagenesis studies of the phosphorylation sites of recombinant human pyruvate dehydrogenase. Site-specific regulation. J. Biol. Chem. 270, 14297–14304. 10.1074/jbc.270.24.14297 7782287

[B113] Korotchkina L. G. Patel M. S. (2001). Site specificity of four pyruvate dehydrogenase kinase isoenzymes toward the three phosphorylation sites of human pyruvate dehydrogenase. J. Biol. Chem. 276, 37223–37229. 10.1074/jbc.M103069200 11486000

[B114] Krowchuk D. P. Frieden I. J. Mancini A. J. Darrow D. H. Blei F. Greene A. K. (2019). Clinical practice guideline for the management of infantile hemangiomas. Pediatrics 143, e20183475. 10.1542/peds.2018-3475 30584062

[B115] Kunimoto K. Yamamoto Y. Jinnin M. (2022). ISSVA classification of vascular anomalies and molecular biology. Int. J. Mol. Sci. 23, 2358. 10.3390/ijms23042358 35216474 PMC8876303

[B116] Lando D. Peet D. J. Gorman J. J. Whelan D. A. Whitelaw M. L. Bruick R. K. (2002). FIH-1 is an asparaginyl hydroxylase enzyme that regulates the transcriptional activity of hypoxia-inducible factor. Genes Dev. 16, 1466–1471. 10.1101/gad.991402 12080085 PMC186346

[B117] Lau M.-T. Klausen C. Leung P. C. K. (2011). E-cadherin inhibits tumor cell growth by suppressing PI3K/Akt signaling via β-catenin-Egr1-mediated PTEN expression. Oncogene 30, 2753–2766. 10.1038/onc.2011.6 21297666

[B118] Le A. Cooper C. R. Gouw A. M. Dinavahi R. Maitra A. Deck L. M. (2010). Inhibition of lactate dehydrogenase A induces oxidative stress and inhibits tumor progression. Proc. Natl. Acad. Sci. U. S. A. 107, 2037–2042. 10.1073/pnas.0914433107 20133848 PMC2836706

[B119] Léauté-Labrèze C. Dumas de la Roque E. Hubiche T. Boralevi F. Thambo J.-B. Taïeb A. (2008). Propranolol for severe hemangiomas of infancy. N. Engl. J. Med. 358, 2649–2651. 10.1056/NEJMc0708819 18550886

[B120] Léauté-Labrèze C. Hoeger P. Mazereeuw-Hautier J. Guibaud L. Baselga E. Posiunas G. (2015). A randomized, controlled trial of oral propranolol in infantile hemangioma. N. Engl. J. Med. 372, 735–746. 10.1056/NEJMoa1404710 25693013

[B121] Lebellec L. Bertucci F. Tresch-Bruneel E. Ray-Coquard I. Le Cesne A. Bompas E. (2018). Prognostic and predictive factors for angiosarcoma patients receiving paclitaxel once weekly plus or minus bevacizumab: an ancillary study derived from a randomized clinical trial. BMC Cancer 18, 963. 10.1186/s12885-018-4828-1 30305054 PMC6180490

[B122] Ledford H. Callaway E. (2019). Biologists who decoded how cells sense oxygen win medicine Nobel. Nature 574, 161–162. 10.1038/d41586-019-02963-0 31595071

[B123] Lee J.-W. Bae S.-H. Jeong J.-W. Kim S.-H. Kim K.-W. (2004). Hypoxia-inducible factor (HIF-1)alpha: its protein stability and biological functions. Exp. Mol. Med. 36, 1–12. 10.1038/emm.2004.1 15031665

[B124] Leek R. D. Talks K. L. Pezzella F. Turley H. Campo L. Brown N. S. (2002). Relation of hypoxia-inducible factor-2 alpha (HIF-2 alpha) expression in tumor-infiltrative macrophages to tumor angiogenesis and the oxidative thymidine phosphorylase pathway in Human breast cancer. Cancer Res. 62, 1326–1329. 11888900

[B125] Leonard B. E. (1972). A comparison between the effects of phenoxybenzamine, phentolamine and propranolol on mouse brain glycolysis. Biochem. Pharmacol. 21, 109–113. 10.1016/0006-2952(72)90256-0 5058699

[B126] Li L. Wu D. Qin X. Mi L.-Z. (2022). PDGF-D prodomain differentially inhibits the biological activities of PDGF-D and PDGF-B. J. Mol. Biol. 434, 167709. 10.1016/j.jmb.2022.167709 35777468

[B127] Li M. Wang X. Yang E. Li Y. Geng Y. Chen Z. (2023). OTUB1 catalytic-independently deubiquitinates TGFBI and mediates the angiogenesis in infantile hemangioma by regulating glycolysis. Arteriosclerosis, Thrombosis, Vasc. Biol. 43, 654–673. 10.1161/ATVBAHA.123.319177 36994729

[B128] Li Y. Zhu X. Li L. Bao C. Liu Q. zhang N. (2024). Construction and applications of the EOMA spheroid model of Kaposiform hemangioendothelioma. J. Biol. Eng. 18, 21. 10.1186/s13036-024-00417-4 38486263 PMC10941415

[B129] Li Z. Yang P. Li Z. (2014). The multifaceted regulation and functions of PKM2 in tumor progression. Biochimica Biophysica Acta (BBA) - Rev. Cancer 1846, 285–296. 10.1016/j.bbcan.2014.07.008 25064846

[B130] Lim Y. H. Bacchiocchi A. Qiu J. Straub R. Bruckner A. Bercovitch L. (2016). GNA14 somatic mutation causes congenital and sporadic vascular tumors by MAPK activation. Am. J. Hum. Genet. 99, 443–450. 10.1016/j.ajhg.2016.06.010 27476652 PMC4974082

[B131] Liu H.-L. Liu D. Ding G.-R. Liao P.-F. Zhang J.-W. (2015). Hypoxia-inducible factor-1α and Wnt/β-catenin signaling pathways promote the invasion of hypoxic gastric cancer cells. Mol. Med. Rep. 12, 3365–3373. 10.3892/mmr.2015.3812 25997455 PMC4526080

[B132] Liu J. Gao Y. Zhang X. (2024). A patent review on hypoxia-inducible factor (HIF) modulators (2021-2023). Expert Opin. Ther. Pat. 34, 651–664. 10.1080/13543776.2024.2368739 38874005

[B133] Liu M. Galli G. Wang Y. Fan Q. Wang Z. Wang X. (2020). Novel therapeutic targets for hypoxia-related cardiovascular diseases: the role of HIF-1. Front. Physiol. 11, 774. 10.3389/fphys.2020.00774 32760290 PMC7375027

[B134] Liu X. Xie P. Hao N. Zhang M. Liu Y. Liu P. (2021). HIF-1–regulated expression of calreticulin promotes breast tumorigenesis and progression through Wnt/β-catenin pathway activation. Proc. Natl. Acad. Sci. U. S. A. 118, e2109144118. 10.1073/pnas.2109144118 34706936 PMC8612225

[B135] Liu X. H. Li J. Y. Qu X. H. Yan W. L. Zhang L. Yang C. (2016). Treatment of kaposiform hemangioendothelioma and tufted angioma. Int. J. Cancer 139, 1658–1666. 10.1002/ijc.30216 27252149

[B136] Long E. Ilie M. Hofman V. Havet K. Selva E. Butori C. (2009). LANA-1, Bcl-2, Mcl-1 and HIF-1alpha protein expression in HIV-associated Kaposi sarcoma. Virchows Arch. 455, 159–170. 10.1007/s00428-009-0791-1 19484260

[B137] López Gutiérrez J. C. Avila L. F. Sosa G. Patron M. (2007). Placental anomalies in children with infantile hemangioma. Pediatr. Dermatol. 24, 353–355. 10.1111/j.1525-1470.2007.00450.x 17845154

[B138] Lorusso B. Cerasoli G. Falco A. Frati C. Graiani G. Madeddu D. (2022). Β-blockers activate autophagy on infantile hemangioma-derived endothelial cells *in vitro* . Vasc. Pharmacol. 146, 107110. 10.1016/j.vph.2022.107110 36103993

[B139] Luo W. Semenza G. L. (2012). Emerging roles of PKM2 in cell metabolism and cancer progression. Trends Endocrinol. Metab. 23, 560–566. 10.1016/j.tem.2012.06.010 22824010 PMC3466350

[B140] Luo Z. Tian M. Yang G. Tan Q. Chen Y. Li G. (2022). Hypoxia signaling in human health and diseases: implications and prospects for therapeutics. Signal Transduct. Target Ther. 7, 218. 10.1038/s41392-022-01080-1 35798726 PMC9261907

[B141] Lyons L. L. North P. E. Mac-Moune Lai F. Stoler M. H. Folpe A. L. Weiss S. W. (2004). Kaposiform hemangioendothelioma: a study of 33 cases emphasizing its pathologic, immunophenotypic, and biologic uniqueness from juvenile hemangioma. Am. J. Surg. Pathol. 28, 559–568. 10.1097/00000478-200405000-00001 15105642

[B142] Ma L. Li G. Zhu H. Dong X. Zhao D. Jiang X. (2014). 2-Methoxyestradiol synergizes with sorafenib to suppress hepatocellular carcinoma by simultaneously dysregulating hypoxia-inducible factor-1 and -2. Cancer Lett. 355, 96–105. 10.1016/j.canlet.2014.09.011 25218350

[B143] Ma T. Jham B. C. Hu J. Friedman E. R. Basile J. R. Molinolo A. (2010). Viral G protein-coupled receptor up-regulates Angiopoietin-like 4 promoting angiogenesis and vascular permeability in Kaposi’s sarcoma. Proc. Natl. Acad. Sci. U. S. A. 107, 14363–14368. 10.1073/pnas.1001065107 20660728 PMC2922606

[B144] Ma T. Patel H. Babapoor-Farrokhran S. Franklin R. Semenza G. L. Sodhi A. (2015). KSHV induces aerobic glycolysis and angiogenesis through HIF-1-dependent upregulation of pyruvate kinase 2 in Kaposi’s sarcoma. Angiogenesis 18, 477–488. 10.1007/s10456-015-9475-4 26092770 PMC4659376

[B145] Maeda-Otsuka S. Kajihara I. Tasaki Y. Yamada-Kanazawa S. Sakamoto R. Sawamura S. (2019). Hypoxia accelerates the progression of angiosarcoma through the regulation of angiosarcoma cells and tumor microenvironment. J. Dermatological Sci. 93, 123–132. 10.1016/j.jdermsci.2019.01.005 30704938

[B146] Magar A. G. Morya V. K. Kwak M. K. Oh J. U. Noh K. C. (2024). A molecular perspective on HIF-1α and angiogenic stimulator networks and their role in solid tumors: an update. Int. J. Mol. Sci. 25, 3313. 10.3390/ijms25063313 38542288 PMC10970012

[B147] Mahon P. C. Hirota K. Semenza G. L. (2001). FIH-1: a novel protein that interacts with HIF-1alpha and VHL to mediate repression of HIF-1 transcriptional activity. Genes Dev. 15, 2675–2686. 10.1101/gad.924501 11641274 PMC312814

[B148] Majmundar A. J. Wong W. J. Simon M. C. (2010). Hypoxia-inducible factors and the response to hypoxic stress. Mol. Cell 40, 294–309. 10.1016/j.molcel.2010.09.022 20965423 PMC3143508

[B149] Makkeyah S. M. Elseedawy M. E. Abdel-Kader H. M. Mokhtar G. M. Ragab I. A. (2022). Vascular endothelial growth factor response with propranolol therapy in patients with infantile hemangioma. Pediatr. Hematol. Oncol. 39, 215–224. 10.1080/08880018.2021.1961956 34477031

[B150] Manalo D. J. Rowan A. Lavoie T. Natarajan L. Kelly B. D. Ye S. Q. (2005). Transcriptional regulation of vascular endothelial cell responses to hypoxia by HIF-1. Blood 105, 659–669. 10.1182/blood-2004-07-2958 15374877

[B151] Mansfield S. A. Williams R. F. Iacobas I. (2020). Vascular tumors. Semin. Pediatr. Surg. 29, 150975. 10.1016/j.sempedsurg.2020.150975 33069294

[B152] Mark R. J. Poen J. C. Tran L. M. Fu Y. S. Juillard G. F. (1996). Angiosarcoma. A report of 67 patients and a review of the literature. Cancer 77, 2400–2406. 10.1002/(SICI)1097-0142(19960601)77:11<2400::AID-CNCR32>3.0.CO;2-Z 8635113

[B153] Marušić Z. Billings S. D. (2017). Histopathology of spindle cell vascular tumors. Surg. Pathol. Clin. 10, 345–366. 10.1016/j.path.2017.01.006 28477885

[B154] Maxwell P. H. Wiesener M. S. Chang G. W. Clifford S. C. Vaux E. C. Cockman M. E. (1999). The tumour suppressor protein VHL targets hypoxia-inducible factors for oxygen-dependent proteolysis. Nature 399, 271–275. 10.1038/20459 10353251

[B155] McGettrick A. F. O’Neill L. A. J. (2020). The role of HIF in immunity and inflammation. Cell Metab. 32, 524–536. 10.1016/j.cmet.2020.08.002 32853548

[B156] Mei H. Xian H. Ke J. (2021). LncRNA-MCM3AP-AS1 promotes the progression of infantile hemangiomas by increasing miR-138-5p/HIF-1α axis-regulated glycolysis. Front. Mol. Biosci. 8, 753218. 10.3389/fmolb.2021.753218 34660700 PMC8511435

[B157] Mitra R. Fitzsimons H. L. Hale T. Tan S. T. Gray C. White M. P. J. (2024). Recent advances in understanding the molecular basis of infantile haemangioma development. Br. J. Dermatol 191, 661–669. 10.1093/bjd/ljae241 38845569

[B158] Malekan M. Ebrahimzadeh MA. Sheida F. (2021). The role of Hypoxia-Inducible Factor-1alpha and its signaling in melanoma. Biomed. & Pharmacother. = Biomedecine & Pharmacother. 141, 111873. 10.1016/j.biopha.2021.111873 34225012

[B159] Morine Y. Shimada M. Utsunomiya T. Imura S. Ikemoto T. Mori H. (2011). Hypoxia inducible factor expression in intrahepatic cholangiocarcinoma. Hepatogastroenterology 58, 1439–1444. 10.5754/hge11156 21940327

[B160] Mou J. Li C. Zheng Q. Meng X. Tang H. (2024). Research progress in tumor angiogenesis and drug resistance in breast cancer. Cancer Biol. Med. 21, 571–585. 10.20892/j.issn.2095-3941.2023.0515 38940663 PMC11271221

[B161] Nagy J. A. Chang S.-H. Shih S.-C. Dvorak A. M. Dvorak H. F. (2010). Heterogeneity of the tumor vasculature. Semin. Thromb. Hemost. 36, 321–331. 10.1055/s-0030-1253454 20490982 PMC3278036

[B162] Nguyen H.-L. Boon L. M. Vikkula M. (2020). Genetics of vascular anomalies. Semin. Pediatr. Surg. 29, 150967. 10.1016/j.sempedsurg.2020.150967 33069286

[B163] Nordsmark M. Alsner J. Keller J. Nielsen O. S. Jensen O. M. Horsman M. R. (2001). Hypoxia in human soft tissue sarcomas: adverse impact on survival and no association with p53 mutations. Br. J. Cancer 84, 1070–1075. 10.1054/bjoc.2001.1728 11308256 PMC2363869

[B164] North P. E. Waner M. Mizeracki A. Mihm M. C. (2000). GLUT1: a newly discovered immunohistochemical marker for juvenile hemangiomas. Hum. Pathol. 31, 11–22. 10.1016/s0046-8177(00)80192-6 10665907

[B165] Ohsawa M. Naka N. Tomita Y. Kawamori D. Kanno H. Aozasa K. (1995). Use of immunohistochemical procedures in diagnosing angiosarcoma. Evaluation of 98 cases. Cancer 75, 2867–2874. 10.1002/1097-0142(19950615)75:12<2867::aid-cncr2820751212>3.0.co;2-8 7773935

[B166] Padera T. P. Stoll B. R. Tooredman J. B. Capen D. di Tomaso E. Jain R. K. (2004). Pathology: cancer cells compress intratumour vessels. Nature 427, 695. 10.1038/427695a 14973470

[B167] Paley P. J. Staskus K. A. Gebhard K. Mohanraj D. Twiggs L. B. Carson L. F. (1997). Vascular endothelial growth factor expression in early stage ovarian carcinoma. Cancer 80, 98–106. 10.1002/(sici)1097-0142(19970701)80:1<98::aid-cncr13>3.0.co;2-a 9210714

[B168] Palsson-McDermott E. M. Curtis A. M. Goel G. Lauterbach M. A. R. Sheedy F. J. Gleeson L. E. (2015). Pyruvate kinase M2 regulates Hif-1α activity and IL-1β induction and is a critical determinant of the warburg effect in LPS-activated macrophages. Cell Metab. 21, 65–80. 10.1016/j.cmet.2014.12.005 25565206 PMC5198835

[B169] Parks S. K. Cormerais Y. Pouysségur J. (2017). Hypoxia and cellular metabolism in tumour pathophysiology. J. Physiol. 595, 2439–2450. 10.1113/JP273309 28074546 PMC5390873

[B170] Pereira E. S. P. Moraes E. T. de Siqueira D. M. Santos M. A. S. dos (2015). Stewart treves syndrome. An Bras Dermatol 90, 229–231. 10.1590/abd1806-4841.20153685 26312725 PMC4540559

[B171] Qiu T. Xiang S. Zhou J. Yang M. Lan Y. Zhang X. (2025). Sirolimus for kaposiform hemangioendothelioma: potential mechanisms of action and resistance. Int. J. Cancer 156, 689–699. 10.1002/ijc.35207 39369447

[B172] Rajakumar T. Pugalendhi P. (2023). Allyl isothiocyanate inhibits invasion and angiogenesis in breast cancer via EGFR-mediated JAK-1/STAT-3 signaling pathway. Amino Acids 55, 981–992. 10.1007/s00726-023-03285-2 37310534

[B173] Rathmell W. K. Acs G. Simon M. C. Vaughn D. J. (2004). HIF transcription factor expression and induction of hypoxic response genes in a retroperitoneal angiosarcoma. Anticancer Res. 24, 167–169. 15015593

[B174] Ritter M. R. Reinisch J. Friedlander S. F. Friedlander M. (2006). Myeloid cells in infantile hemangioma. Am. J. Pathol. 168, 621–628. 10.2353/ajpath.2006.050618 16436675 PMC1606494

[B175] Rodríguez Bandera A. I. Sebaratnam D. F. Wargon O. Wong L.-C. F. (2021). Infantile hemangioma. Part 1: epidemiology, pathogenesis, clinical presentation and assessment. J. Am. Acad. Dermatol 85, 1379–1392. 10.1016/j.jaad.2021.08.019 34419524

[B176] Ronchi A. Cozzolino I. Zito Marino F. De Chiara A. Argenziano G. Moscarella E. (2020). Primary and secondary cutaneous angiosarcoma: distinctive clinical, pathological and molecular features. Annals Diagnostic Pathology 48, 151597. 10.1016/j.anndiagpath.2020.151597 32829071

[B177] Rong Y. Durden D. L. Van Meir E. G. Brat D. J. (2006). Pseudopalisading’ necrosis in glioblastoma: a familiar morphologic feature that links vascular pathology, hypoxia, and angiogenesis. J. Neuropathology & Exp. Neurology 65, 529–539. 10.1097/00005072-200606000-00001 16783163

[B178] Saito M. Gunji Y. Kashii Y. Odaka J. Yamauchi T. Kanai N. (2009). Refractory kaposiform hemangioendothelioma that expressed vascular endothelial growth factor receptor (VEGFR)-2 and VEGFR-3: a case report. J. Pediatr. Hematology/Oncology 31, 194–197. 10.1097/MPH.0b013e3181979c83 19262246

[B179] Salceda S. Caro J. (1997). Hypoxia-inducible factor 1alpha (HIF-1alpha) protein is rapidly degraded by the ubiquitin-proteasome system under normoxic conditions. Its stabilization by hypoxia depends on redox-induced changes. J. Biol. Chem. 272, 22642–22647. 10.1074/jbc.272.36.22642 9278421

[B180] Sang N. Stiehl D. P. Bohensky J. Leshchinsky I. Srinivas V. Caro J. (2003). MAPK signaling up-regulates the activity of hypoxia-inducible factors by its effects on p300. J. Biol. Chem. 278, 14013–14019. 10.1074/jbc.M209702200 12588875 PMC4518846

[B181] Schneider J. W. Dittmer D. P. (2017). Diagnosis and treatment of kaposi sarcoma. Am. J. Clin. Dermatol 18, 529–539. 10.1007/s40257-017-0270-4 28324233 PMC5509489

[B104] Schödel J Grampp S Maher ER Moch H Ratcliffe PJ Russo P Mole DR (2016). Hypoxia, hypoxia-inducible transcription factors, and renal cancer. Eur. Urol. 69, 646–657. 10.1016/j.eururo.2015.08.007 26298207 PMC5012644

[B182] Sebaratnam D. F. Rodríguez Bandera A. L. Wong L.-C. F. Wargon O. (2021). Infantile hemangioma. Part 2: management. J. Am. Acad. Dermatol 85, 1395–1404. 10.1016/j.jaad.2021.08.020 34419523

[B183] Sebestyén A. Kopper L. Dankó T. Tímár J. (2021). Hypoxia signaling in cancer: from basics to clinical practice. Pathol. Oncol. Res. 27, 1609802. 10.3389/pore.2021.1609802 34257622 PMC8262153

[B184] Semenza G. L. (2003). Targeting HIF-1 for cancer therapy. Nat. Rev. Cancer 3, 721–732. 10.1038/nrc1187 13130303

[B185] Semenza G. L. (2019). Pharmacologic targeting of hypoxia-inducible factors. Annu. Rev. Pharmacol. Toxicol. 59, 379–403. 10.1146/annurev-pharmtox-010818-021637 30625281

[B186] Semenza G. L. Jiang B. H. Leung S. W. Passantino R. Concordet J. P. Maire P. (1996). Hypoxia response elements in the aldolase A, enolase 1, and lactate dehydrogenase A gene promoters contain essential binding sites for hypoxia-inducible factor 1. J. Biol. Chem. 271, 32529–32537. 10.1074/jbc.271.51.32529 8955077

[B187] Semenza G. L. Nejfelt M. K. Chi S. M. Antonarakis S. E. (1991). Hypoxia-inducible nuclear factors bind to an enhancer element located 3’ to the human erythropoietin gene. Proc. Natl. Acad. Sci. U. S. A. 88, 5680–5684. 10.1073/pnas.88.13.5680 2062846 PMC51941

[B188] Seto T. Higashiyama M. Funai H. Imamura F. Uematsu K. Seki N. (2006). Prognostic value of expression of vascular endothelial growth factor and its flt-1 and KDR receptors in stage I non-small-cell lung cancer. Lung Cancer 53, 91–96. 10.1016/j.lungcan.2006.02.009 16697074

[B189] Shimobayashi M. Hall M. N. (2014). Making new contacts: the mTOR network in metabolism and signalling crosstalk. Nat. Rev. Mol. Cell Biol. 15, 155–162. 10.1038/nrm3757 24556838

[B190] Shin Y. C. Joo C.-H. Gack M. U. Lee H.-R. Jung J. U. (2008). Kaposi’s sarcoma-associated herpesvirus viral IFN regulatory factor 3 stabilizes hypoxia-inducible factor-1 alpha to induce vascular endothelial growth factor expression. Cancer Res. 68, 1751–1759. 10.1158/0008-5472.CAN-07-2766 18339855

[B191] Shrestha P. Davis D. A. Veeranna R. P. Carey R. F. Viollet C. Yarchoan R. (2017). Hypoxia-inducible factor-1 alpha as a therapeutic target for primary effusion lymphoma. PLoS Pathog. 13, e1006628. 10.1371/journal.ppat.1006628 28922425 PMC5619862

[B192] Siegel J. H. Janis R. Alper J. C. Schutte H. Robbins L. Blaufox M. D. (1969). Disseminated visceral Kaposi’s sarcoma. Appearance after human renal homograft operation. JAMA 207, 1493–1496. 10.1001/jama.1969.03150210077009 4884743

[B193] Singh R. K. Bose D. Robertson E. S. (2022). Epigenetic reprogramming of Kaposi’s sarcoma-associated herpesvirus during hypoxic reactivation. Cancers (Basel) 14, 5396. 10.3390/cancers14215396 36358814 PMC9654037

[B194] Smeland E. Kilvaer T. K. Sorbye S. Valkov A. Andersen S. Bremnes R. M. (2012). Prognostic impacts of hypoxic markers in soft tissue sarcoma. Sarcoma 2012, 541650. 10.1155/2012/541650 22454562 PMC3289941

[B195] Sodhi A. Chaisuparat R. Hu J. Ramsdell A. K. Manning B. D. Sausville E. A. (2006). The TSC2/mTOR pathway drives endothelial cell transformation induced by the Kaposi’s sarcoma-associated herpesvirus G protein-coupled receptor. Cancer Cell 10, 133–143. 10.1016/j.ccr.2006.05.026 16904612

[B196] Steelman L. S. Bertrand F. E. McCubrey J. A. (2004). The complexity of PTEN: mutation, marker and potential target for therapeutic intervention. Expert Opin. Ther. Targets 8, 537–550. 10.1517/14728222.8.6.537 15584861

[B197] Stefan D. C. (2015). Patterns of distribution of childhood cancer in Africa. J. Trop. Pediatr. 61, 165–173. 10.1093/tropej/fmv005 25724211

[B198] Swain S. M. Shastry M. Hamilton E. (2023). Targeting HER2-positive breast cancer: advances and future directions. Nat. Rev. Drug Discov. 22, 101–126. 10.1038/s41573-022-00579-0 36344672 PMC9640784

[B199] Tan E. M. S. Chudakova D. A. Davis P. F. Brasch H. D. Itinteang T. Tan S. T. (2015). Characterisation of subpopulations of myeloid cells in infantile haemangioma. J. Clin. Pathol. 68, 571–574. 10.1136/jclinpath-2014-202846 25834091

[B200] Tanaka T. Wiesener M. Bernhardt W. Eckardt K.-U. Warnecke C. (2009). The human HIF (hypoxia-inducible factor)-3alpha gene is a HIF-1 target gene and may modulate hypoxic gene induction. Biochem. J. 424, 143–151. 10.1042/BJ20090120 19694616

[B201] Tang Y.-A. Chen Y.-F. Bao Y. Mahara S. Yatim S. M. J. M. Oguz G. (2018). Hypoxic tumor microenvironment activates GLI2 via HIF-1α and TGF-β2 to promote chemoresistance in colorectal cancer. Proc. Natl. Acad. Sci. U. S. A. 115, E5990–E5999. 10.1073/pnas.1801348115 29891662 PMC6042102

[B202] Theodoropoulos V. E. Lazaris A. C. Kastriotis I. Spiliadi C. Theodoropoulos G. E. Tsoukala V. (2005). Evaluation of hypoxia-inducible factor 1alpha overexpression as a predictor of tumour recurrence and progression in superficial urothelial bladder carcinoma. BJU Int. 95, 425–431. 10.1111/j.1464-410X.2005.05314.x 15679808

[B203] Theodoropoulos V. E. Lazaris A. C. Sofras F. Gerzelis I. Tsoukala V. Ghikonti I. (2004). Hypoxia-inducible factor 1 alpha expression correlates with angiogenesis and unfavorable prognosis in bladder cancer. Eur. Urol. 46, 200–208. 10.1016/j.eururo.2004.04.008 15245814

[B204] Tortorelli I. Bellan E. Chiusole B. Murtas F. Ruggieri P. Pala E. (2024). Primary vascular tumors of bone: a comprehensive literature review on classification, diagnosis and treatment. Crit. Rev. Oncology/Hematology 195, 104268. 10.1016/j.critrevonc.2024.104268 38237880

[B205] Tu R. Kang W. Yang M. Wang L. Bao Q. Chen Z. (2021). USP29 coordinates MYC and HIF1α stabilization to promote tumor metabolism and progression. Oncogene 40, 6417–6429. 10.1038/s41388-021-02031-w 34601505

[B206] Urban M. J. Williams E. F. (2024). Vascular lesions. Facial Plast. Surg. Clin. North Am. 32, 13–25. 10.1016/j.fsc.2023.09.003 37981409

[B207] Vageli D. P. Doukas P. G. Goupou K. Benos A. D. Astara K. Zacharouli K. (2024). Hypoxia-inducible factor 1alpha and vascular endothelial growth factor in Glioblastoma Multiforme: a systematic review going beyond pathologic implications. Oncol. Res. 32, 1239–1256. 10.32604/or.2024.052130 39055895 PMC11267112

[B208] Vander Heiden M. G. Cantley L. C. Thompson C. B. (2009). Understanding the Warburg effect: the metabolic requirements of cell proliferation. Science 324, 1029–1033. 10.1126/science.1160809 19460998 PMC2849637

[B209] van Vugt L. J. van der Vleuten C. J. M. Flucke U. Blokx W. A. M. (2017). The utility of GLUT1 as a diagnostic marker in cutaneous vascular anomalies: a review of literature and recommendations for daily practice. Pathology - Res. Pract. 213, 591–597. 10.1016/j.prp.2017.04.023 28552538

[B210] Vaupel P. Fortmeyer H. P. Runkel S. Kallinowski F. (1987). Blood flow, oxygen consumption, and tissue oxygenation of human breast cancer xenografts in nude rats. Cancer Res. 47, 3496–3503. 3581084

[B211] Vaupel P. Höckel M. Mayer A. (2007). Detection and characterization of tumor hypoxia using pO2 histography. Antioxid. Redox Signal 9, 1221–1235. 10.1089/ars.2007.1628 17536958

[B212] Vaupel P. Mayer A. Briest S. Höckel M. (2003). Oxygenation gain factor: a novel parameter characterizing the association between hemoglobin level and the oxygenation status of breast cancers. Cancer Res. 63, 7634–7637. 14633681

[B213] Vaupel P. Schlenger K. Knoop C. Höckel M. (1991). Oxygenation of human tumors: evaluation of tissue oxygen distribution in breast cancers by computerized O2 tension measurements. Cancer Res. 51, 3316–3322. 2040005

[B214] Virtanen A. Pukkala E. Auvinen A. (2007). Angiosarcoma after radiotherapy: a cohort study of 332,163 Finnish cancer patients. Br. J. Cancer 97, 115–117. 10.1038/sj.bjc.6603805 17519906 PMC2359658

[B215] Wan J. Wu W. (2016). Hyperthermia induced HIF-1a expression of lung cancer through AKT and ERK signaling pathways. J. Exp. Clin. Cancer Res. 35, 119. 10.1186/s13046-016-0399-7 27456341 PMC4960890

[B216] Wang G. Sweren E. Andrews W. Li Y. Chen J. Xue Y. (2023). Commensal microbiome promotes hair follicle regeneration by inducing keratinocyte HIF-1α signaling and glutamine metabolism. Sci. Adv. 9, eabo7555. 10.1126/sciadv.abo7555 36598999 PMC9812389

[B217] Wang G. L. Jiang B. H. Rue E. A. Semenza G. L. (1995). Hypoxia-inducible factor 1 is a basic-helix-loop-helix-PAS heterodimer regulated by cellular O2 tension. Proc. Natl. Acad. Sci. U. S. A. 92, 5510–5514. 10.1073/pnas.92.12.5510 7539918 PMC41725

[B218] Wang G. L. Semenza G. L. (1995). Purification and characterization of hypoxia-inducible factor 1. J. Biol. Chem. 270, 1230–1237. 10.1074/jbc.270.3.1230 7836384

[B219] Wang H. Kaplan F. S. Pignolo R. J. (2024). The HIF-1α and mTOR pathways amplify heterotopic ossification. Biomolecules 14, 147. 10.3390/biom14020147 38397384 PMC10887042

[B220] Wang J.-C. Li G.-Y. Wang B. Han S.-X. Sun X. Jiang Y.-N. (2019a). Metformin inhibits metastatic breast cancer progression and improves chemosensitivity by inducing vessel normalization via PDGF-B downregulation. J. Exp. Clin. Cancer Res. 38, 235. 10.1186/s13046-019-1211-2 31164151 PMC6549289

[B221] Wang Z. Yao W. Sun H. Dong K. Ma Y. Chen L. (2019b). Sirolimus therapy for kaposiform hemangioendothelioma with long-term follow-up. J. Dermatol 46, 956–961. 10.1111/1346-8138.15076 31489702

[B222] Wang Z. Zheng C. Sun H. Yao W. Li K. Ma Y. (2020). Immunohistochemical analysis of mTOR pathway-related proteins in kaposiform hemangioendothelioma. Dermatology 236, 262–270. 10.1159/000503604 31896113

[B223] Warburg O. (1925). The metabolism of carcinoma cells. J. Cancer Res. 9, 148–163. 10.1158/jcr.1925.148

[B224] Watari K. Shibata T. Fujita H. Shinoda A. Murakami Y. Abe H. (2020). NDRG1 activates VEGF-A-induced angiogenesis through PLCγ1/ERK signaling in mouse vascular endothelial cells. Commun. Biol. 3, 107. 10.1038/s42003-020-0829-0 32144393 PMC7060337

[B225] Wei L. Li L. Xu Z. Zhang B. Han X. Wang C. (2022). Comparison of effectiveness of two different doses of propranolol on kaposiform hemangioendothelioma. Front. Pediatr. 10, 760401. 10.3389/fped.2022.760401 35419320 PMC8996134

[B226] Wicks E. E. Semenza G. L. (2022). Hypoxia-inducible factors: cancer progression and clinical translation. J. Clin. Invest 132, e159839. 10.1172/JCI159839 35642641 PMC9151701

[B227] Wiesener M. S. Jürgensen J. S. Rosenberger C. Scholze C. K. Hörstrup J. H. Warnecke C. (2003). Widespread hypoxia-inducible expression of HIF-2alpha in distinct cell populations of different organs. FASEB J. 17, 271–273. 10.1096/fj.02-0445fje 12490539

[B228] Wu D. Potluri N. Lu J. Kim Y. Rastinejad F. (2015). Structural integration in hypoxia-inducible factors. Nature 524, 303–308. 10.1038/nature14883 26245371

[B229] Wu M. Chen L. Qi Y. Ci H. Mou S. Yang J. (2022). Human umbilical cord mesenchymal stem cell promotes angiogenesis via integrin β1/ERK1/2/HIF-1α/VEGF-A signaling pathway for off-the-shelf breast tissue engineering. Stem Cell Res. Ther. 13, 99. 10.1186/s13287-022-02770-x 35255978 PMC8900416

[B230] Wu P. Xu H. Li N. Huo R. Shen B. Lin X. (2021). Hypoxia-induced Cyr61/CCN1 production in infantile hemangioma. Plast. Reconstr. Surg. 147, 412e–423e. 10.1097/PRS.0000000000007672 33587560

[B231] Xia H. Zhu J. Wang J. Ren J. Cai Y. Wang F. (2017). Association of ATF4 expression with tissue hypoxia and M2 macrophage infiltration in infantile hemangioma. J. Histochem Cytochem 65, 285–294. 10.1369/0022155417694872 28438094 PMC5407535

[B232] Xiang S. Gong X. Qiu T. Zhou J. Yang K. Lan Y. (2024). Insights into the mechanisms of angiogenesis in infantile hemangioma. Biomed. Pharmacother. 178, 117181. 10.1016/j.biopha.2024.117181 39059349

[B233] Xiao Y. Yu D. (2021). Tumor microenvironment as a therapeutic target in cancer. Pharmacol. Ther. 221, 107753. 10.1016/j.pharmthera.2020.107753 33259885 PMC8084948

[B234] Xin H. Zhong C. Nudleman E. Ferrara N. (2016). Evidence for pro-angiogenic functions of VEGF-ax. Cell 167, 275–284.e6. 10.1016/j.cell.2016.08.054 27662093

[B235] Xu Q. Briggs J. Park S. Niu G. Kortylewski M. Zhang S. (2005). Targeting Stat3 blocks both HIF-1 and VEGF expression induced by multiple oncogenic growth signaling pathways. Oncogene 24, 5552–5560. 10.1038/sj.onc.1208719 16007214

[B236] Yang J. Xu J. Wang W. Zhang B. Yu X. Shi S. (2023). Epigenetic regulation in the tumor microenvironment: molecular mechanisms and therapeutic targets. Sig Transduct. Target Ther. 8, 210. 10.1038/s41392-023-01480-x PMC10203321 37217462

[B237] Yang S.-L. Wu C. Xiong Z.-F. Fang X. (2015). Progress on hypoxia-inducible factor-3: its structure, gene regulation and biological function (Review). Mol. Med. Rep. 12, 2411–2416. 10.3892/mmr.2015.3689 25936862

[B238] Young R. J. Brown N. J. Reed M. W. Hughes D. Woll P. J. (2010). Angiosarcoma. Lancet Oncol. 11, 983–991. 10.1016/S1470-2045(10)70023-1 20537949

[B239] Young R. J. Woll P. J. Staton C. A. Reed M. W. R. Brown N. J. (2014). Vascular-targeted agents for the treatment of angiosarcoma. Cancer Chemother. Pharmacol. 73, 259–270. 10.1007/s00280-013-2345-0 24253175

[B240] Yu X. Li W. Deng Q. You S. Liu H. Peng S. (2017a). Neoalbaconol inhibits angiogenesis and tumor growth by suppressing EGFR-mediated VEGF production. Mol. Carcinog. 56, 1414–1426. 10.1002/mc.22602 27996164

[B241] Yu X. Zhang J. Wu Z. Liu M. Chen R. Gu Y. (2017b). LUMBAR syndrome: a case manifesting as cutaneous infantile hemangiomas of the lower extremity, perineum and gluteal region, and a review of published work. J. Dermatol 44, 808–812. 10.1111/1346-8138.13763 28191659

[B242] Zhang P. Yao Q. Lu L. Li Y. Chen P.-J. Duan C. (2014). Hypoxia-inducible factor 3 is an oxygen-dependent transcription activator and regulates a distinct transcriptional response to hypoxia. Cell Rep. 6, 1110–1121. 10.1016/j.celrep.2014.02.011 24613356

[B243] Zhang W. Sun L. Gao H. Wang S. (2023). Mechanism of the HIF-1α/VEGF/VEGFR-2 pathway in the proliferation and apoptosis of human haemangioma endothelial cells. Int. J. Exp. Pathology 104, 258–268. 10.1111/iep.12485 PMC10500167 37381118

[B244] Zhao F.-L. Qin C.-F. (2019). EGF promotes HIF-1α expression in colorectal cancer cells and tumor metastasis by regulating phosphorylation of STAT3. Eur. Rev. Med. Pharmacol. Sci. 23, 1055–1062. 10.26355/eurrev_201902_16993 30779072

[B245] Zhao X. Tang Y.-P. Wang C.-Y. Wu J.-X. Ye F. (2019). Prognostic values of STAT3 and HIF-1α in esophageal squamous cell carcinoma. Eur. Rev. Med. Pharmacol. Sci. 23, 3351–3357. 10.26355/eurrev_201904_17698 31081089

[B246] Zhao Y. Xing C. Deng Y. Ye C. Peng H. (2024). HIF-1α signaling: essential roles in tumorigenesis and implications in targeted therapies. Genes Dis. 11, 234–251. 10.1016/j.gendis.2023.02.039 37588219 PMC10425810

[B247] Zhou J. Lan F. Liu M. Wang F. Ning X. Yang H. (2024). Hypoxia inducible factor-1ɑ as a potential therapeutic target for osteosarcoma metastasis. Front. Pharmacol. 15, 1350187. 10.3389/fphar.2024.1350187 38327979 PMC10847273

[B248] Zukerberg L. R. Nickoloff B. J. Weiss S. W. (1993). Kaposiform hemangioendothelioma of infancy and childhood. An aggressive neoplasm associated with Kasabach-Merritt syndrome and lymphangiomatosis. Am. J. Surg. Pathol. 17, 321–328. 10.1097/00000478-199304000-00001 8494101

